# Semaglutide and pioglitazone restore airway mucosal homeostasis by resolving *Pseudomonas aeruginosa* pyocyanin-induced mucus hypersecretion, ciliostasis, and type 2 airway inflammation

**DOI:** 10.3389/fimmu.2026.1877061

**Published:** 2026-09-07

**Authors:** Cong Wu, Nitish A. Kulkarni, Shi Qian Lew, Woosuk Choi, Mayandi Sivaguru, Beata Kosmider, Som G. Nanjappa, Gee W. Lau

**Affiliations:** 1 Department of Pathobiology, University of Illinois Urbana-Champaign, Urbana, IL, United States; 2 Cytometry and Microscopy to Omics (CMtO) Facility, Roy J. Carver Biotechnology Center, University of Illinois Urbana-Champaign, Urbana, IL, United States; 3 Earth Science and Environmental Change, School of Earth, Society and the Environment, University of Illinois Urbana-Champaign, Urbana, IL, United States; 4 Center for Inflammation and Lung Research, Department of Microbiology, Immunology and Inflammation, Lewis Katz School of Medicine, Temple University, Philadelphia, PA, United States

**Keywords:** lung homeostasis, pioglitazone, proinflammatory responses, pyocyanin, semaglutide

## Abstract

**Rationale:**

Chronic obstructive pulmonary disease (COPD) is the third leading cause of death worldwide. As lung transplantation is the only curative option and current therapies are largely based on managing clinical symptoms, novel treatments are urgently needed.

**Objective:**

Previously, we showed that the first-generation incretin Exedine-4 could attenuate airway mucus dysregulation induced by the *Pseudomonas aeruginosa* redox-active toxin pyocyanin by modulating GLP1R-PPARγ signaling. In this study, we compared the efficacy of currently approved next-generation GLP1R agonists and PPARγ agonists in improving airway mucus homeostasis, mucociliary escalator function, and *P*. *aeruginosa* clearance after chronic exposure to pyocyanin, as well as the underlying mechanisms.

**Methods:**

Six GLP-1R agonists and two PPARγ agonists were compared based on their ability to attenuate pyocyanin-induced oxidative stress and mucin overexpression in 16HBE14o− cells by the cellular reactive oxygen species (ROS) assays, immunoblotting, and confocal immunofluorescence microscopy. The ability of these agonists to restore the mucociliary escalator function in air-liquid interface (ALI) cultures of primary human small airway epithelial cells (SAECs) was captured by confocal immunofluorescence microscopy. Then, we examined whether these agonists could restore mucus homeostasis, neutralize lung proinflammatory responses, and reduce bacterial burden in C57BL/6 mice. Finally, the expression of relevant pro- and anti-mucin biosynthesis signaling pathways was investigated.

**Measurements and main results:**

Semaglutide and Pioglitazone are the most effective in decreasing the expression of mucus biomarker MUC5AC mucin and ROS production in response to pyocyanin, both *in vitro* and *in vivo*. Additionally, these agonists restore mucociliary beat frequency and mucociliary transport impaired by pyocyanin. The protective effects of these drugs were partially abolished by respective antagonists, Exendin(9-39) and GW9662, against GLP1R and PPARγ, confirming the GLP1R signaling pathway-dependent mechanisms. Moreover, Semaglutide and Pioglitazone also attenuated type 2 immune responses and neutrophil influx, both of which are known drivers of mucus hypersecretion, and reduced the *P. aeruginosa* burden in a chronic bronchitis model of infection in mouse lungs.

**Conclusions:**

Our findings identify a promising adjunctive therapeutic avenue for COPD by repurposing FDA-approved Semaglutide and Pioglitazone, which are readily available for clinical use.

## 1 Introduction

Chronic obstructive pulmonary disease (COPD) is a chronic disease that deteriorates lung function as a result of aberrant remodeling in the small airways and alveolar space, causing 3 million deaths worldwide annually (1, 2). The etiologies of COPD, among others, include cigarette smoking, air pollutants, and particulates from wildfire and biomass burning as major causes of the disease (3). Prolonged exposure to these environmental irritants causes a chronic inflammatory response in the airways and alveolar epithelium remodeling, leading to chronic bronchitis and emphysema. A major pathological feature of COPD is airway obstruction caused by mucus hypersecretion and failure in clearance, which creates a niche favorable to chronic microbial colonization and infection. *Pseudomonas aeruginosa* (PA) is one of the major pathogens in patients with advanced stages of COPD (4). PA expresses a plethora of virulence factors, among which, pyocyanin (PCN), a potent redox-cycling toxin, plays a critical virulence role in both mouse models of acute and chronic pneumonia (5–7). Current COPD therapies focus primarily on symptom management and improving quality of life through smoking cessation, oxygen therapy, vaccination against pathogens, and PDE4 inhibitors (8). Therefore, alternative approaches are urgently needed to supplement current COPD management.

Mucociliary clearance (MCC) is a critical component of the pulmonary innate defense system that traps inhaled particles and toxins in mucus and eliminates them through the coordinated action of the mucociliary escalator function (9, 10). Mucus is produced by goblet cells and submucosal glands. Airway mucus homeostasis is tightly regulated by Forkhead box protein A2 (FOXA2), a key transcriptional repressor of airway mucin glycoprotein biosynthesis genes, including *MUC5AC* and *MUC5B*, encoding the two predominant mucins in the airway mucus layer (11–14). We and others have shown that activation of IL4/IL13-STAT6-SPDEF and EGFR-PI3K-AKT/MEK1/2-ERK1/2 signaling pathways convergently inhibit FOXA2 expression, leading to goblet cell metaplasia and hyperplasia and airway mucus overproduction (13–16). In chronically diseased lungs, aberrant epithelial tethering of mucins and excessive mucus burden impede the mucociliary clearance (MCC) (11, 12, 17). Previously, we have shown that PCN activates both IL4/IL13-STAT6-SPDEF and EGFR-PI3K-AKT/MEK1/2-ERK1/2 signaling pathways to inhibit FOXA2 expression, which exacerbates airway mucus hypersecretion (18–21), highlighting the synergetic pathological effects caused by inflammatory conditions and microbial infection in COPD.

COPD is often comorbid with type 2 diabetes mellitus (T2DM) (22–24). Therefore, careful monitoring and managing blood glucose levels are critically important in these patients. Incretin mimetics, including the prototypic Exendin-4 (Ex-4), are synthetic analogs of glucagon-like peptide-1 (GLP-1) approved by the FDA for the treatment of T2DM (25, 26). Ex-4 binds to the G protein-coupled glucagon-like peptide-1 receptor (GLP1R) to activate downstream pathways including cAMP-PKA, PPARγ, MAPK, B-RAF, and TORC2, which initiate exocytosis, insulin biosynthesis, beta cell proliferation, and neogenesis. FOXA2 is activated and serves as a transcription factor in these cellular processes. Similarly, thiazolidinediones (TZDs) have been widely used to manage T2DM (27). Rosiglitazone, a TZD and PPARγ agonist, has been shown to increase FOXA2 expression in β-cells (28). In an *in vitro* model of mucin production by the immortalized human lung mucoepidermoid carcinoma NCI-H292 cells’ airway mucin production, Rosiglitazone repressed *MUC5AC* transcription by promoting the localization of PPARγ to the peroxisome proliferator-activated receptor response element (PPRE) within the *MUC5AC* gene (29). Similarly, incretin mimetics have enhanced resistance to dipeptidyl peptidase-4 (DPP-4)-mediated degradation, and GLP1R-mediated signaling upregulates FOXA2 and PPARγ expression in mouse pancreatic cells, restoring β-cell biomass, insulin secretion, and sensitivity (30–32).

Based on an initial report that Ex-4 increases FOXA2 expression and binding to the promoters of target genes in liver cells (33), we have recently examined the synergistic regulatory effects of GLP1R- and PPARγ-mediated signaling pathways on FOXA2 expression. We repurposed Ex-4 and Troglitazone, a PPARγ agonist, as novel mucoregulators to attenuate mucus hypersecretion. Our data indicate that Ex-4 and Troglitazone restore airway mucus homeostasis through the GLP1R-PKA-PPARγ-FOXA2/tyrosine phosphatases axis in *P.aeruginosa*-infected mouse lungs and in normal and chronically diseased human primary airway epithelial cells exposed to PCN (19). Activated PTEN and PTP1B phosphatases dephosphorylate and inactivate key kinases within the pro-goblet cell transdifferentiation IL4/IL13-STAT6-SPDEF and EGFR-PI3K-AKT/MEK1/2-ERK1/2 signaling pathways and restore the expression of FOXA2 to suppress mucus overexpression induced by PCN in diseased airway epithelial cells (14, 19).

Despite its regulatory effects on airway mucin expression in experimental models, Troglitazone was withdrawn from the market due to severe hepatotoxicity (34). Rosiglitazone has been associated with cardiovascular toxicity (35). Recently, however, Pioglitazone, a newer generation of TZD, has emerged as a more viable candidate because it offers improved metabolic profiles, including the suppression of cholesterol synthesis, and a significantly favorable safety profile compared to the first-generation TZDs (27, 34). Also, the main second-generation GLP1R agonists are engineered with distinct pharmacokinetic properties, allowing for a diverse range of dosing regimens. These are broadly classified into short-acting (Exenatide twice daily, Lixisenatide, and oral Semaglutide once daily) and long-acting drugs (Exenatide, Dulaglutide, Liraglutide, and Semaglutide once weekly) (36). These pharmacological advancements may widen the clinical utility of GLP1R agonists in patients with chronic respiratory diseases, potentially mitigating adverse effects while maximizing therapeutic efficacy. However, despite their improved safety and efficacy, it remains to be determined whether these new T2DM therapeutics can effectively attenuate goblet cell differentiation and mucin expression in the context of chronic airway diseases, as we have previously demonstrated for Ex-4 and Troglitazone (19). In this study, we investigated the mucoactive effects of six incretin mimetics (Ex-4, Liraglutide, Lixisenatide, Dulaglutide, Semaglutide, and Albiglutide) and two second-generation TZDs (Pioglitazone and Rosiglitazone) in PCN-exposed airway epithelial cells.

## 2 Materials and methods

### 2.1 Chemicals and reagents

All the GLP1R and PPARγ agonists were purchased from MedChemExpress: Exendin-4 (HY-13443), Liraglutide (HY-P0014), Lixisenatide (HY-P0119), Dulaglutide (HY-P0120), Semaglutide (HY-114118), Albiglutide (HY-108795), Rosiglitazone (HY-17386), and Pioglitazone (HY-13956). The GLP1R antagonist Exendin 9-39 (HY-P0264) was also purchased from MedChemExpress. The PPARγ antagonist GW9662 (M6191) was purchased from Sigma. For the preparation of working solutions, these compounds were resuspended in DMSO, 5% acetic acid, PBS, or sterile water (1 mM or 1mg/mL) according to the manufacturer’s recommendation. Pyocyanin (P0046, Sigma) was aliquoted for frozen stocks at -20 °C after being dissolved in DMSO.

### 2.2 Antibodies

For primary antibodies, except for MUC5AC (E9V10, Cell Signaling Technology, for immunofluorescence labeling), the following antibodies were purchased from Santa Cruz Biotechnology: MUC5AC (sc-21701) for Western blotting and FOXA2 (sc-374376). HRP-conjugated secondary antibodies were purchased from Santa Cruz Biotechnology: anti-mouse IgG HRP-conjugated (sc-516102) and mouse anti-rabbit HRP-conjugated (sc-2357). Fluorescent-conjugated secondary antibodies were purchased from Invitrogen: anti-mouse IgG conjugated to Alexa Fluor 594 (A21203) and anti-rabbit IgG conjugated to Alexa Fluor 647 (A31573). Antibodies were diluted and used according to the manufacturer’s recommendation; further refinement is needed.

### 2.3 Submerged and air-liquid interface cell cultures

Frozen stocks of 16HBE14o- human bronchial epithelial cells were seeded on plates precoated with a type I bovine collagen solution (5005, Advanced BioMatrix) and cultured in a submerged manner using the Minimum Essential Medium (MEM, 10-009-CV, Corning). For expansion and passaging, the cells were cultured in a submerged manner. To passage the cells, cells were treated with 0.05% trypsin (25-052-Cl, Corning) and seeded on plates without coating beyond passage 3 using MEM supplemented with 10% fetal bovine serum (FBS) and 1% penicillin-streptomycin. Cells at passage 3 or later were used for all functional experiments, except ciliary motility measurements. For the primary human small airway epithelial cells (SAECs) (CC-2547, Lonza), frozen stocks were seeded as described for 16HBE14o- and cultured in submerged fashion until passages 2–3 by using the Small Airway Cell Basal Medium (SABM, CC-3119, Lonza) with SAGM SingleQuots^®^ Supplement Pack (CC-4124, Lonza).

To develop ALI cultures with pseudostratified airway epithelium, both 16HBE14o- and SAECs from the submerged cultures were seeded onto Costar Transwell inserts (3460, Corning), precoated with Human Collagen Type IV (C7521, Sigma), and cultured in submerged conditions with SABM medium for 5 days. On day 6, the media in the transwell were aspirated, and the media in the bottom chambers were replaced with complete PheumoCult-ALI medium (05001, Stemcell) containing the PheumoCult-ALI supplement (05003, Stemcell), heparin (07890, Stemcell), and hydrocortisone (07925, Stemcell). Airway cells were allowed to polarize for 4 weeks before exposure to PCN for 24 h with or without drug treatment.

### 2.4 Measurement of cellular ROS

ROS levels in PCN-exposed 16HBE14o- cells were determined using the Cellular Reactive Oxygen Species Assay kit (ab186027, Abcam) according to the manufacturer’s guidelines. 16HBE14o- cells were pretreated with or without incretin mimetics or TZDs for 60 minutes, followed by another 60 minutes of exposure to PCN (20 µl of 55 µg/ml). The fluorescence level (Excitation/Emission: 520/605) was measured with a SpectraMax iD5 (Molecular Devices).

### 2.5 Western blot analyses

Whole-cell lysates were prepared from airway epithelial cells using MPER Mammalian Protein Extraction Reagent (78501, Thermo Fisher Scientific), and immunoblotting was performed as described previously described (19, 20, 37), with modifications. Briefly, the M-PER Mammalian Protein Extraction Reagent was used to extract proteins from the airway cells. Protein concentrations were quantified by using the Pierce BCA Protein Assay Kits (23225, Thermo Fisher Scientific). Except for mucin proteins, all the target proteins were separated by using 12% SDS-PAGE resolving non-gradient gels. For mucin Western blotting, gradient agarose-acrylamide hybrid gels were used to separate the large glycoproteins (>250 kDa), as we have previously published (19). Proteins were transferred onto a 0.45 µm nitrocellulose membrane (88018, Thermo Fisher Scientific), followed by blocking with 5% skim milk, overnight incubation at 4 °C with specific primary antibodies, and visualized with HRP-conjugated secondary antibodies. The chemiluminescent signal was developed by SuperSignal™ West Femto Maximum Sensitivity Substrate (34096, Thermo Fisher Scientific). All antibodies used in Western blot analysis were diluted according to the manufacturer’s recommendations.

### 2.6 Immunofluorescence staining of ALI cultures

The membrane of the Transwell insert was excised and incubated in 4% paraformaldehyde (J19943.K2, Thermo Fisher Scientific) at 4 °C overnight. Then, the cells were permeabilized in PBST with 0.1% Triton X (H5141, Promega) for 45 minutes, followed by blocking with PBST containing 5% FBS for 1 hour. The membranes were incubated overnight at 4 °C with primary antibodies diluted in blocking solution. To probe the primary antibodies, the membranes were incubated with Alexa Fluor-conjugated secondary antibodies at room temperature for 1 hour, followed by DAPI staining for 15 minutes. The membranes were mounted with Fluoromount-G (0100-01, Southern Biotech) and visualized by using a high-resolution confocal microscope (Nikon, Eclipse Ti). All antibodies were diluted according to the manufacturer’s recommendations.

### 2.7 Mucociliary transport experiment

To measure cilia motility, FluoSpheres™ Polystyrene Microspheres (F13081, Invitrogen) were diluted to desirable concentrations and added to the apical surface of ALI-cultured SAECs. Live imaging of the microsphere’s motion was recorded for 1 minute per sample using a Zeiss LSM 980 microscope and further analyzed in Imaris (Oxford Instruments, version 10.2.0) using the Spot module.

### 2.8 Mouse study approval

Mouse experiments were conducted in strict compliance with the guidelines outlined in the “Guide for the Care and Use of Laboratory Animals” by the National Institutes of Health. All experimental procedures were approved by the Institutional Animal Care and Use Committee (IACUC) at the University of Illinois at Urbana-Champaign under Protocols 20120 and 23132.

### 2.9 Mouse model of PCN exposure with semaglutide and pioglitazone treatment for immune cells analyses

Six-week-old C57BL/6 mice (cohorts of 8, sex-matched) were purchased from Charles River Inc. and housed in positively ventilated microisolator cages equipped with automatic recirculating water in a room with laminar, high-efficiency particle-accumulation-filtered air, autoclaved food, water, and bedding. Mice were subcutaneously injected once daily with Semaglutide (160 µg/kg) and Pioglitazone (165 µg/kg), followed by intranasal instillation of 37.5 µg of PCN (in 50 µl) once daily for 2 weeks. Control cohorts were subcutaneously injected with 50 µl sterile PBS. At the end of 2 weeks, some cohorts of mice were euthanized by exposure to 5% isoflurane, followed by cervical dislocation and bilateral thoracotomy, and lungs were inflated and fixed with 10% neutral buffered formalin for histopathological, immunohistochemical, and immunofluorescence analysis. For other cohorts, bronchoalveolar lavage fluids (BALF) were collected for immune cell analysis.

### 2.10 Mouse model of PCN exposure with semaglutide and pioglitazone treatment for histopathological analysis and *P. aeruginosa* infection

C57BL/6 mice (cohorts of 20) were subcutaneously treated once daily with Semaglutide (160 µg/kg) and Pioglitazone (165 µg/kg), followed by intranasal instillation of 25 µg of PCN (in 50 µl) once daily for 3 weeks. Control cohorts were subcutaneously injected with 50 µl sterile PBS. At the end of 3 weeks, some mice were used for histopathological analysis (PAS and IHC staining, see below). The remaining mice were intranasally infected with 10^6^ CFU *P. aeruginosa* strain PAO1 on Days 1, 3, and 5. On Day 7, mice were euthanized by exposure to 5% isoflurane, followed by cervical dislocation and bilateral thoracotomy. Bacterial burden was determined by serial dilution plating of lung homogenates.

### 2.11 *In vitro* assays of *P. aeruginosa* killing by semaglutide and pioglitazone

Overnight stationary phase cells of *P. aeruginosa* strain PAO1 were pelleted at 13,200 rpm for 1 minute and washed three times in sterile PBS. PAO1 bacteria were diluted to 10^5–^10^6^ colony forming units (CFUs)/mL of sterile PBS, MEM, Luria Broth, or Mueller-Hinton Broth, before addition of and treated immediately with PBS vs. Semaglutide (1 uM) vs. Pioglitazone (10 uM) in 24-well cell culture plates. Plates were incubated for 3 hours in 37°C with 5% CO2. The extent of bacterial killing was determined by serial dilution plating on the *Pseudomonas* Isolation Agar.

### 2.12 PAS and IHC staining

After mouse euthanasia, a cannula was inserted into the trachea, and the lungs were inflated with 10% neutral-buffered formalin at a constant pressure. The trachea was then ligated, and the inflated lungs were kept in 10% neutral-buffered formalin for 24 hours prior to paraffin embedding. For PAS staining, 5-micrometer paraffin-embedded mouse lung sections were stained with PAS reagent (395B, Sigma) according to the manufacturer’s protocol. For IHC, mouse lung and human sections were stained with primary antibodies against FOXA2/MUC5AC and visualized with the VECTASTAIN Elite ABC-HRP kit (Peroxidase, PK-6101, Vector Laboratories).

### 2.13 Control and COPD lung tissues

Paraffin-embedded control lung tissue sections from deidentified donors whose lungs were not suitable for transplantation and donated for medical research by the Gift of Life Foundation were provided by Dr. Beata Kosmider. Lung tissue from COPD transplants was also obtained. The study was conducted in accordance with the Declaration of Helsinki. The Institutional Review Board (IRB) at Temple University has designated this research as a non-human subject (IRB protocol #23201).

### 2.14 Sex as a biological variable

This study examined male and female mice, and similar findings are reported for both sexes.

### 2.15 Flow cytometry

Antibodies were purchased from Biolegend, BD Biosciences, and eBioscience. Single-cell suspensions from the lungs and bronchoalveolar lavage (BAL) were filtered using 40μm BD Cell Strainers (352340, Corning). Red blood cells were lysed with 4% ammonium chloride in 1X PBS. Cells were first incubated with anti-FCγ receptor MAbs (Fc Block, 553141, BD Biosciences) for 10 minutes on ice. Next, the cells were incubated with fluorophore-conjugated antibodies for surface markers CD4, Thy1.2, CD3e, CD44, and Live/Dead stain (L34961, ThermoFisher) in FACS buffer (2% BSA in 1X PBS with 0.1% NaN3) for 30 minutes on ice. The neutrophils were gated among non-Thy1.2+ cells based on the expression of surface markers CD11b and Ly6G. To determine cytokine production by T cells, the cells were re-stimulated with PMA (P1585, 5 ng/mL; Sigma-Aldrich) and ionomycin (500 ng/mL; 73722, Stemcell Technologies) in the presence of Golgi Stop (554724, BD Biosciences) at 37 °C for 4 h. After 4 h, cells were stained with fluorophore-conjugated antibodies, anti-IL-4 (17-7041-82, ThermoFisher), anti-IL-13 (61-7133-82, ThermoFisher), anti-IL-17A (AF647, Cat. No.560184, BD Biosciences), and anti-IFNγ (AF700, Cat. No.557998, BD Biosciences) for surface markers and then washed, fixed with Fix/Perm buffer (554714, BD Biosciences) for 15–20 minutes on ice. Thereafter, stained with antibodies for intracellular cytokines in Perm/Wash buffer (554723, BD Biosciences) for 30–45 minutes on ice. The cells were analyzed by an Aurora spectral flow cytometer (Cytek). The data were analyzed with FlowJo v10.10 (BD Biosciences).

### 2.16 Statistical analysis and software

Statistical analyses were conducted using GraphPad Prism 10 software and are presented as the mean ± s.e.m. by one-way ANOVA followed by Tukey’s multiple comparisons test. Significance levels were shown as: *p < 0.05, **p < 0.01, ***p < 0.001, ****p < 0.0001.

## 3 Results

### 3.1 Incretin mimetics and PPARγ agonists effectively attenuate ROS production induced by PCN

**Figure 1:**
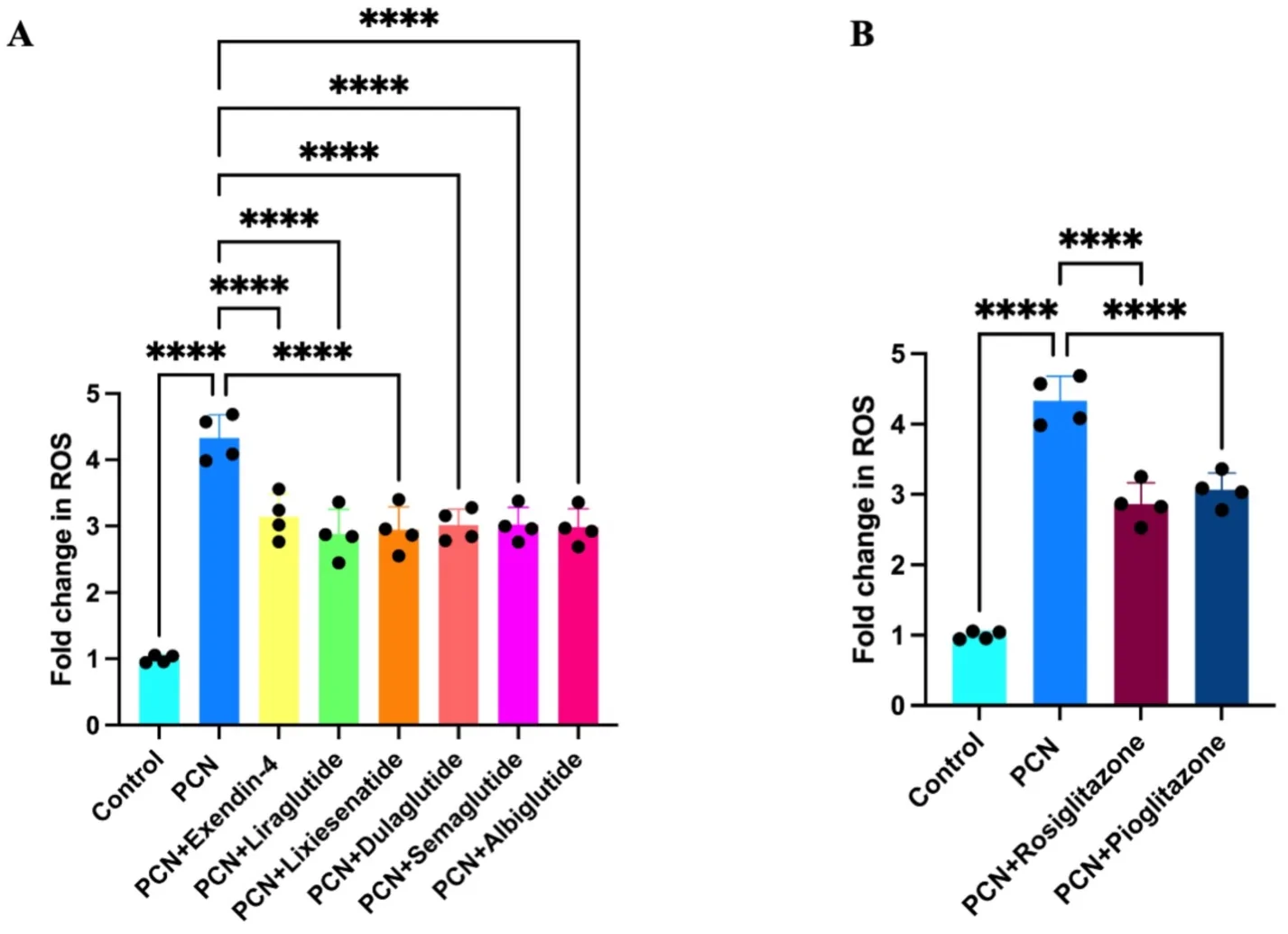
GLP1R and PPARγ agonists are effective in decreasing ROS production induced by PCN. Confluent, submerged 16HBEo- cells were pretreated with 6 GLP1R agonists (1 µM) and 2 PPARγ agonists (10 µM) for 1 hour. Then, cells were treated with 55 µg/mL PCN (in 20 µL/well) for 1 hour, and the intracellular ROS was measured using an excitation/emission at 520/605 nm in bottom-read mode using the Cellular Reactive Oxygen Species Assay kit (ab186027, Abcam) according to the manufacturer’s instructions. **(A)** All 6 GLP1R agonists effectively attenuate the intracellular ROS levels induced by PCN. **(B)** Both PPARγ agonists lowered intracellular ROS levels induced by PCN. The experiments were performed in duplicate and independently four times. All data are presented as mean ± s.e.m. from all 4 experiments. ****p < 0.0001 as analyzed by one-way ANOVA followed by Tukey’s multiple comparisons test.

Pyocyanin is a redox-cycling toxin produced by *P.* aeruginosa that supports its colonization and aggravates acute exacerbations in COPD patients (5, 6). Pyocyanin markedly elevates intracellular ROS and reactive nitrogen species (RNS) levels in airway epithelial cells, thereby disrupting antimicrobial defense mechanisms and ATP production, facilitating bacterial persistence and contributing to chronic lung inflammation in COPD (5, 6). Because our previous findings showed that EX-4 and Troglitazone suppress PCN-induced airway mucus hypersecretion (19), we compared the effectiveness of newer-generation GLP1R agonists and TZDs in limiting PCN-mediated airway pathologies. Due to the FDA withdrawal, Troglitazone was excluded from our analysis. We first measured the intracellular ROS levels in PCN-exposed 16HBE14o- cells with and without drug treatment. GLP1R and PPARγ agonists significantly reduced PCN-induced intracellular ROS levels in 16HBE14o- cells (**Figures 1A, B**). Interestingly, no significant differences in antioxidative capacity were observed across the various agonists.

### 3.2 Semaglutide and Pioglitazone exhibit the strongest suppression of PCN-induced MUC5AC expression in pyocyanin-exposed submerged 16HBE14o- cells

**Figure 2:**
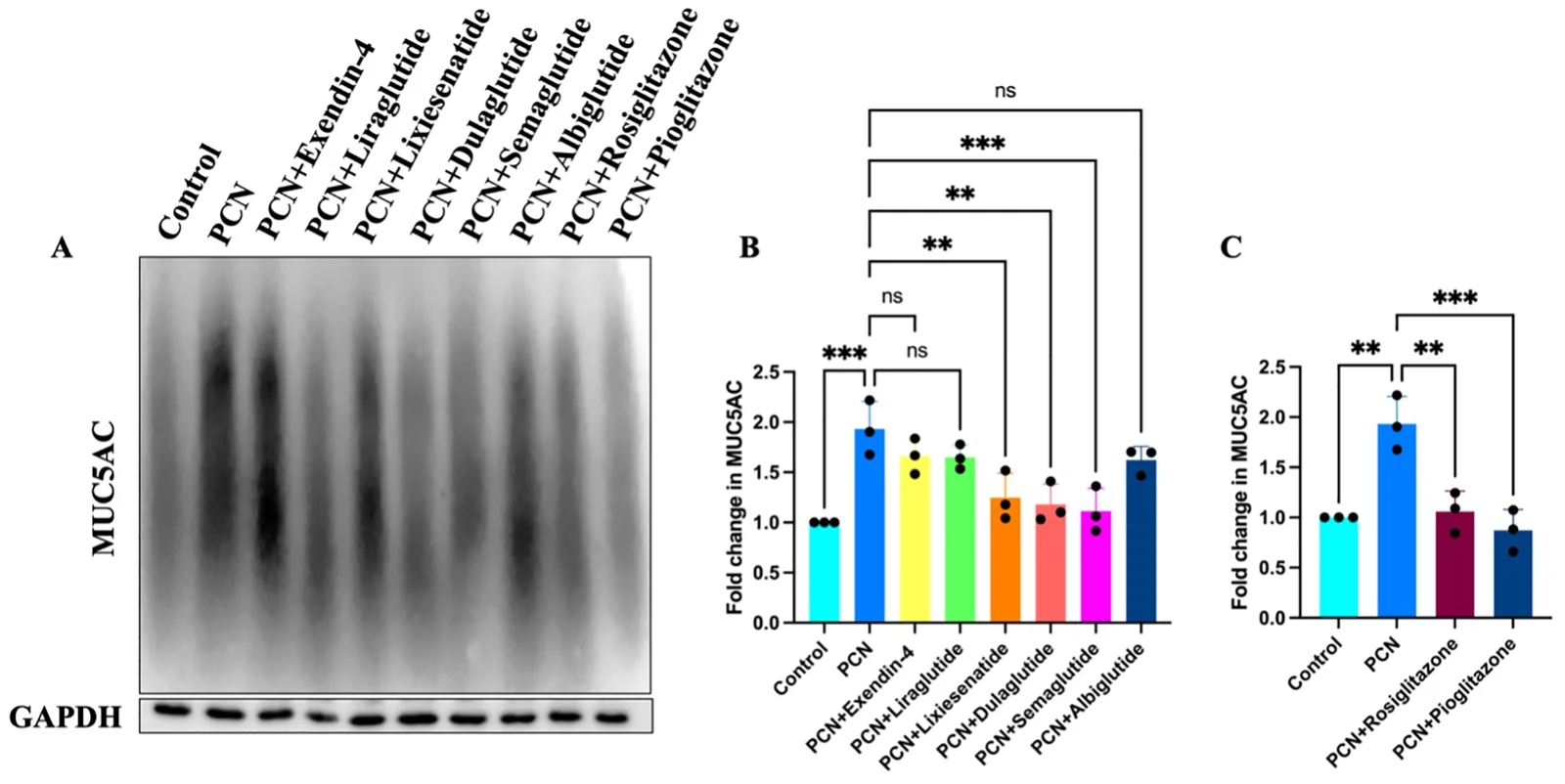
Semaglutide and pioglitazone are most effective in suppressing mucin production induced by PCN in submerged 16HBEo- cells. Confluent submerged 16HBEo- cells were pretreated with each of the 6 GLP1R agonists (1 µM) and 2 PPARγ agonists (10 µM), respectively, for 1 hour before exposure to PCN (5 µg/mL) for 24 hours. **(A)** Western blot analysis for all 8 agonists’ efficacy to attenuate the expression of MUC5AC mucin induced by PCN. The experiments were performed independently three times in triplicate. Additional MUC5AC western blots can be found in **Supplementary Figure 1**. **(B, C)** Densitometric quantification of western blot in A from the three replicates. All data are presented as mean ± s.e.m. from 3 experiments. Ns = no significance, **p < 0.01, and ***p < 0.001, as analyzed by one-way ANOVA followed by Tukey’s multiple comparisons test.

To further distinguish the efficacy of incretin mimetics and TZDs in suppressing mucus hypersecretion, we examined their mucoregulatory capacity by measuring MUC5AC protein abundance. Similar to the reduction in ROS levels, MUC5AC expression was significantly reduced in GLP1R- and PPARγ-agonist-treated groups compared to the PCN-exposed group, except for Albiglutide (**Figures 2A, B**, **S1**). Notably, among the drugs, Semaglutide and Pioglitazone exhibited the most potent suppression of PCN-induced mucin overproduction (**Figures 2A–C**, **S1**).

### 3.3 Semaglutide and Pioglitazone exhibit the strongest restoration of FOXA2 and suppression of MUC5AC expression in the PCN-exposed air-liquid interface culture of 16HBE14o- cells

**Figure 3:**
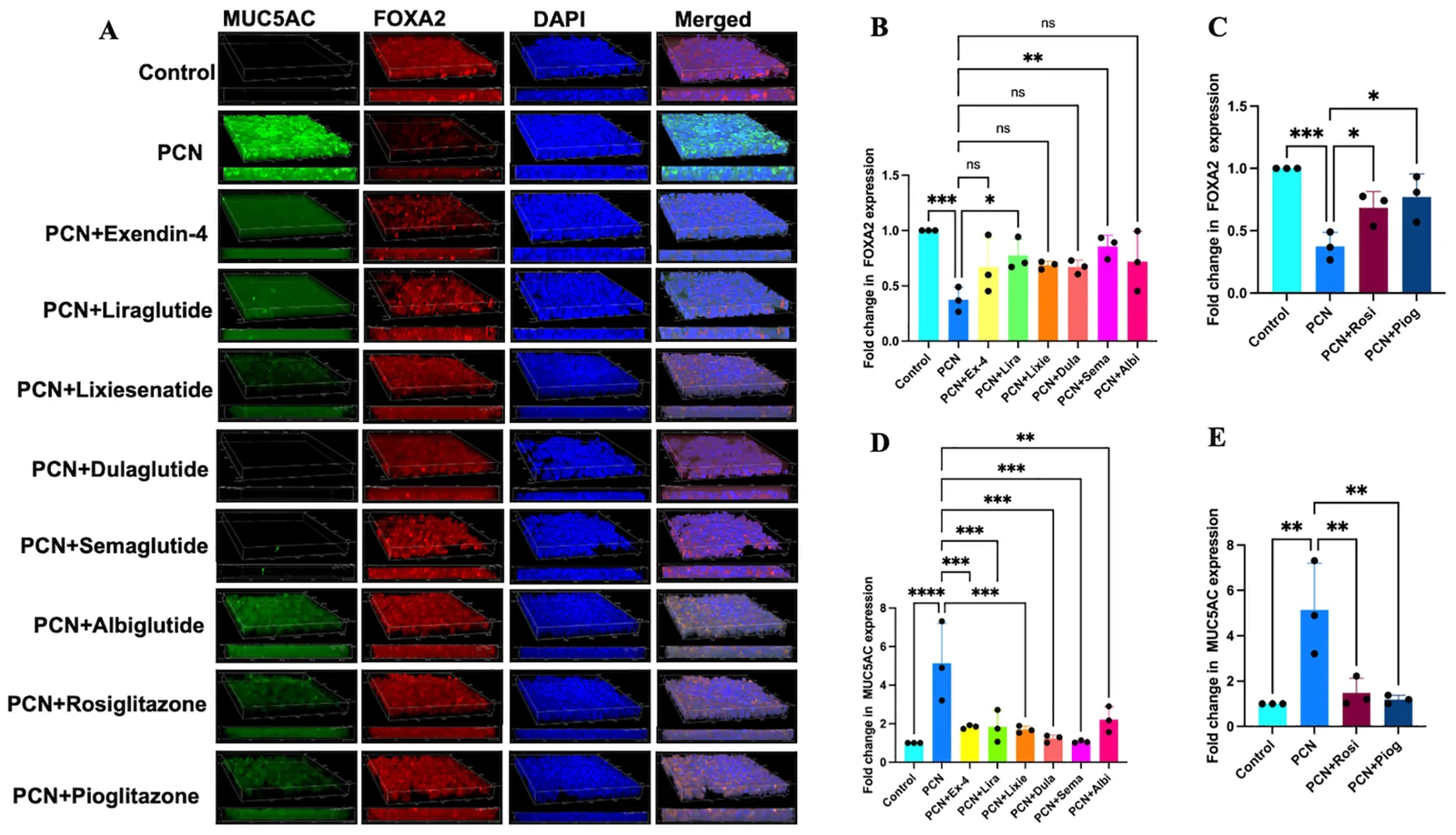
Semaglutide and pioglitazone exhibit the best efficacy in restoring FOXA2 function and in attenuating MUC5AC mucin expression in the air-liquid interface (ALI) culture of 16HBEo- cells exposed to pyocyanin. Partially-differentiated ALI cultured 16HBEo- cells (28 days after air lift) were pretreated for 1 hour with individual GLP1R agonists (1 µM) or PPARγ agonists (10 µM), followed by potentiation by PCN (5 µg/mL) for 24 hours. **(A)** Images of ALI cultures stained with primary antibodies and visualized with Alexa Fluor 488 (green) or Alexa Fluor 594 (red)-conjugated secondary antibodies. Additional images are shown in **Supplementary Figure 2**. **(B, C)** Quantification of fluorescence intensity of FOXA2 in **(A)**. **(D, E)** Quantification of fluorescence intensity of MUC5AC in **(A)** The experiments were performed independently three times. The images were captured using a Nikon Eclipse Ti-S Microscope, and fluorescence density was quantified using ImageJ. All data are presented as mean ± s.e.m. from 3 experiments. **p < 0.01, ***p < 0.001, ****p < 0.0001, ns = no significance, as analyzed by one-way ANOVA followed by Tukey’s multiple comparisons test.

The epithelial lining of the human upper respiratory tract is a pseudostratified structure comprised of multiple cell types, including basal cells, secretory club cells, ciliated cells, goblet cells, and neuroendocrine cells. Air-liquid interface (ALI) culture is a 3D *in vitro* method where airway epithelial cells are cultured on porous membranes fed by culture medium on their basal side with their apical side exposed to air, which triggers differentiation into a functional, mucociliary, pseudostratified layer, more accurately recapitulating human airways than the submerged cell culture. We used the ALI cultures of 16HBE14o- cells to examine the ability of various incretin mimetics and PPARγ agonists in attenuating PCN-induced MUC5AC expression. In line with the results presented in **Figure 2**, confocal immunofluorescence imaging revealed that PCN exposure significantly decreased FOXA2 expression relative to control, accompanied by overexpression of MUC5AC (**Figures 3A, D**, **S2**). GLP1R agonists partially restored FOXA2 levels, with Semaglutide demonstrating the most pronounced recovery among the incretin mimetics (**Figures 3A, B**). Additionally, Pioglitazone showed a stronger ability than Rosiglitazone to restore FXOA2 expression (**Figures 3A, C**, **S2**). For most of the GLP1R agonists, even though the restoration of FOXA2 expression did not reach statistically significant levels (**Figure 3B**), the amount of increased FOXA2 expression was adequate to markedly reduce MUC5AC levels, with Semaglutide showing the most significant inhibitory effect (**Figure 3D**). Similarly, among the TZDs tested, Pioglitazone produced a greater reduction in PCN-induced MUC5AC expression (**Figure 3E**). Collectively, these findings indicate that multiple GLP1R and PPARγ agonists attenuate PCN-induced mucus dysregulation. Among the compounds evaluated, Semaglutide and Pioglitazone consistently demonstrated the strongest overall restoration of FOXA2 expression and suppression of MUC5AC overproduction across both submerged and ALI culture systems. Therefore, these two clinically relevant agonists were selected as representative lead compounds for the subsequent mechanistic studies.

### 3.4 Semaglutide and Pioglitazone suppress mucin overproduction through reestablishing the GLP1R-PKA-PPARγ-FOXA2-phosphatases pathways that inhibit IL4/IL13-STAT6-SPDEF and EGFR-PI3K-AKT/MEK1/2-ERK1/2 signaling in pyocyanin-exposed airway epithelium

**Figure 4:**
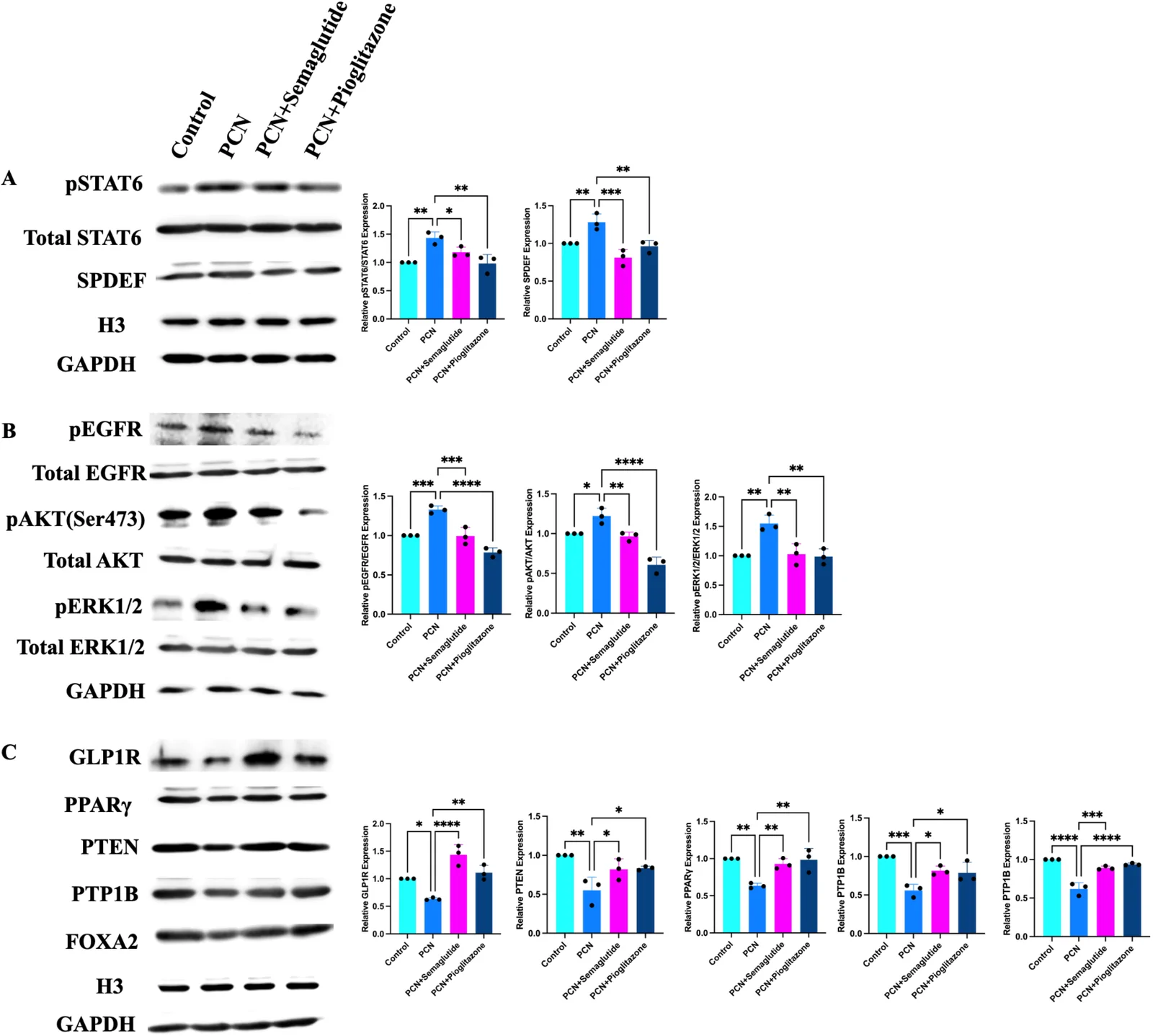
Semaglutide and pioglitazone attenuate the mucus through reestablishing the GLP1R-PKA-PPARγ-FOXA2-phosphatase signaling. Submerged 16HBEo- cells were pretreated with semaglutide (1 µM) and pioglitazone (10 µM) for 1 hour, followed by exposure to 5 µg/mL PCN for 24 hours. **(A)** Semaglutide and pioglitazone attenuated PCN-induced STAT6 phosphorylation and SPDEF expression. **(B)** Semaglutide and pioglitazone markedly inhibit the pEGFR, pAKT, and pERK1/2 expression. **(C)** Semaglutide and pioglitazone restore the expression of GLP1R, PPARγ, FOXA2, and phosphatases PTEN and PTP1B. (**(A–C)** right panels) Densitometric quantification of pSTAT6/STAT6, SPDEF, pEGFR/EGFR, pAKT/AKT, pERK1/2/ERK1/2, GLP1R, PPARγ, PTEN, PTP1B, and FOXA2. Phosphorylated proteins were normalized to their corresponding total proteins, whereas total protein expression was normalized to GAPDH or histone H3 as appropriate. Protein expression is presented relative to the control group (set to 1.0). The experiments were performed independently three times. Additional images are shown in **Supplementary Figure 3**. Data represent the mean ± SEM from three independent experiments. Statistical analysis was performed using one-way ANOVA followed by Tukey’s multiple comparisons test. *P < 0.05, **P < 0.01, ***P < 0.001, ****P < 0.0001.

As discussed earlier, FOXA2 is a key transcriptional factor that maintains airway mucus homeostasis. Two major signaling pathways, IL4/IL13-STAT6-SPDEF and EGFR-PI3K-AKT/MEK1/2-ERK1/2, negatively regulate FOXA2 activity and stability, thereby promoting goblet cell differentiation. Given that Semaglutide and Pioglitazone treatment significantly attenuated the PCN-induced MUC5AC mucin expression, we investigated whether these two drugs restore FOXA2 expression and mucus homeostasis by inhibiting EGFR–AKT/ERK1/2 and STAT6–SPDEF signaling pathways. Western Blot analysis demonstrated that Semaglutide and Pioglitazone treatment attenuated STAT6 phosphorylation and SPDEF expression induced by PCN (**Figure 4A**, **S3A–B**). Consistently, Densitometric analysis confirmed a significant reduction in the pSTAT6/STAT6 ratio and SPDEF protein expression following treatment with either agonist (**Figure 4A**). Likewise, PCN-induced phosphorylation of EGFR, AKT, and ERK1/2 was markedly inhibited by Semaglutide and Pioglitazone, as demonstrated by representative immunoblots (**Figure 4B**, **S3C–D**), and quantitative densitometric analyses of the pEGFR/EGFR, pAKT/AKT, and pERK1/2/ERK1/2 ratios (**Figure 4B**).

Next, we determined whether the restoration of FOXA2 expression was mediated by both PTEN and PTP1B phosphatases downstream of the GLP1R–PKA–PPARγ signaling pathway. As shown in **Figure 4C**, PCN exposure significantly reduced the expression of GLP1R, PPARγ, FOXA2, PTEN, and PTP1B, whereas treatment with Semaglutide or Pioglitazone restored the expression of all five proteins (**Figure 4C**, **S3E–F**). These findings are consistent with our previous study demonstrating that activation of the GLP1R–PPARγ signaling axis upregulates PTEN and PTP1B, leading to dephosphorylation of EGFR, STAT6, AKT, and ERK1/2 and subsequent restoration of FOXA2 activity and mucus homeostasis. Together, these data indicate that Semaglutide and Pioglitazone suppress PCN-induced mucin overproduction by reestablishing the GLP1R–PPARγ–FOXA2–phosphatase signaling axis.

### 3.5 Semaglutide and Pioglitazone restore mucociliary transport inhibited by pyocyanin

**Figure 5:**
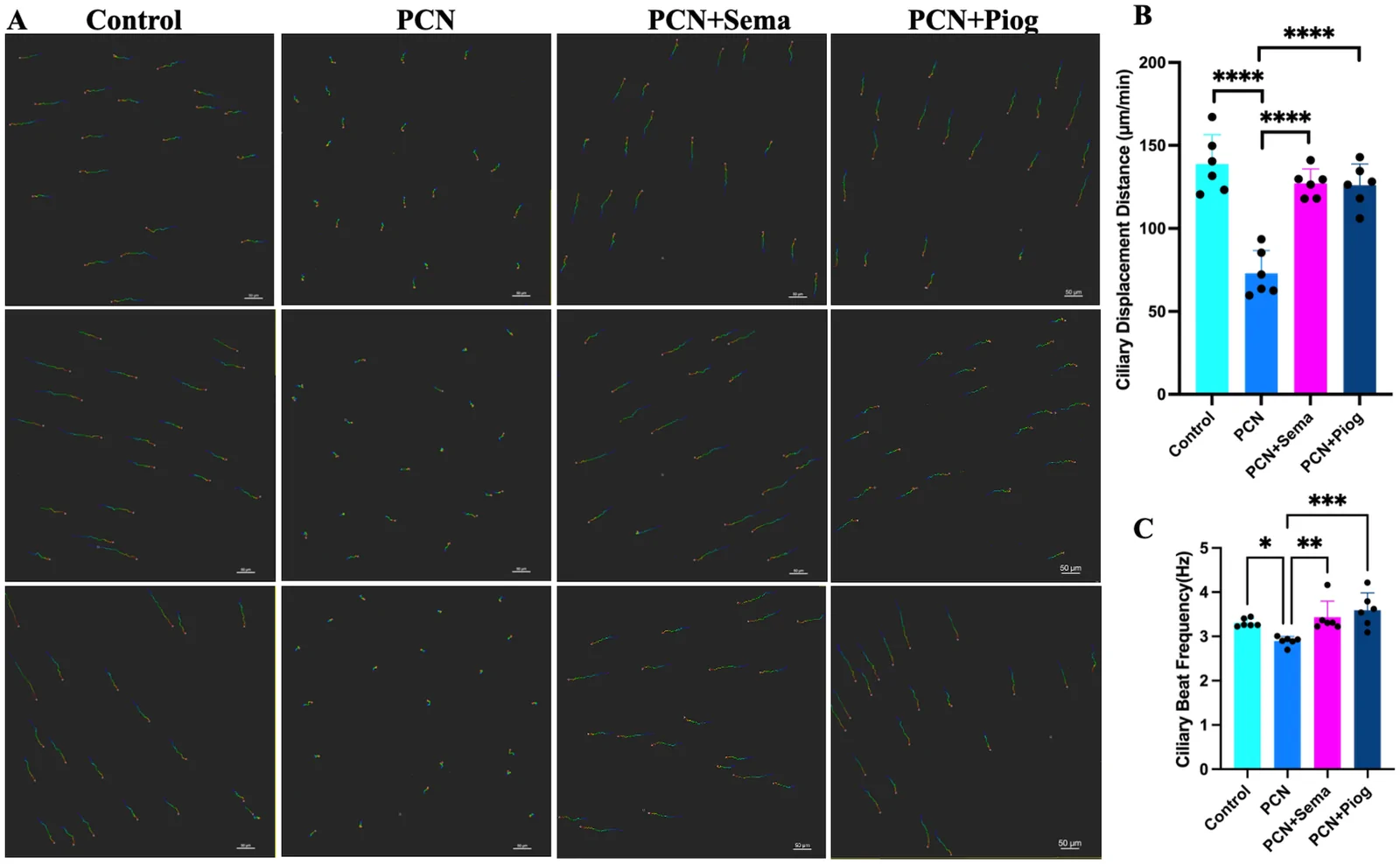
Semaglutide and pioglitazone restore ciliary beat frequency (CBF) and ciliary displacement distance (CDD) disrupted by pyocyanin. The overall experimental scheme is provided in **Supplementary Figure 4**. ALI cultures were pretreated with semaglutide (Sema, 1 µM) and pioglitazone (Piog, 10 µM) for 1 hour, followed by 24 hours of exposure to 5 µg/mL PCN. Then, FITC-labeled fluorescent beads were added to the apical surface of the ALI cultures, and CDD and CBF were tracked and recorded using a Zeiss LSM 980 confocal microscope for 1 minute for each sample and further analyzed in Imaris (Oxford Instruments, version 10.2.0) using the Spot module. **(A)** Magnified representative tracks of the beads’ movement and their distance. The experiments were performed independently twice in triplicate. Additional images at lower magnification are shown in **Supplementary Figure 5**. **(B, C)** Qualification of the CDD and CBF. Each dot represents one independent replicate, and each replicate has ~ 200 to 400 FITC-labeled beads. All data are presented as mean ± s.e.m. from two experiments. *p < 0.05, **p < 0.01, ***p < 0.001, ****p < 0.0001 as analyzed by one-way ANOVA followed by Tukey’s multiple comparisons test.

Because Semaglutide and Pioglitazone reduced mucin production induced by PCN, we hypothesized that their treatment might improve mucociliary clearance. To investigate changes in mucociliary clearance dynamics following PCN exposure and subsequent treatment with the agents, we monitored mucus flow by tracking the displacement of fluorescence-conjugated micro-latex beads in ALI-cultured human primary small airway epithelial cells (SAECs) (**Supplementary Figure 4**). We found that mucus transport velocity was markedly attenuated by PCN; however, this impairment was significantly reversed by Semaglutide and Pioglitazone treatment (**Figure 5A**, **S5**). To further evaluate whether Semaglutide and Pioglitazone improve the mucus flow by restoring ciliary function, we quantified ciliary beat frequency (CBF) and ciliary displacement distance (CDD). CBF refers to the number of effective ciliary strokes per second, and CDD represents the amplitude or effective excursion of ciliary movement during each beat cycle (38). Both CBF and CDD were significantly impaired by PCN but were importantly restored by Semaglutide and Pioglitazone (**Figures 5B, C**). Lower magnification images with can be found in **Supplementary Figure 4**. Collectively, our data suggest that in addition to restoring FOXA2 expression and function and attenuating mucus expression, Semaglutide and Pioglitazone could ameliorate airway mucus stasis by improving mucociliary escalator function in PCN-exposed airway epithelium.

### 3.6 GLP1R and PPARγ antagonists partially abolish the protective effects of semaglutide and pioglitazone

**Figure 6:**
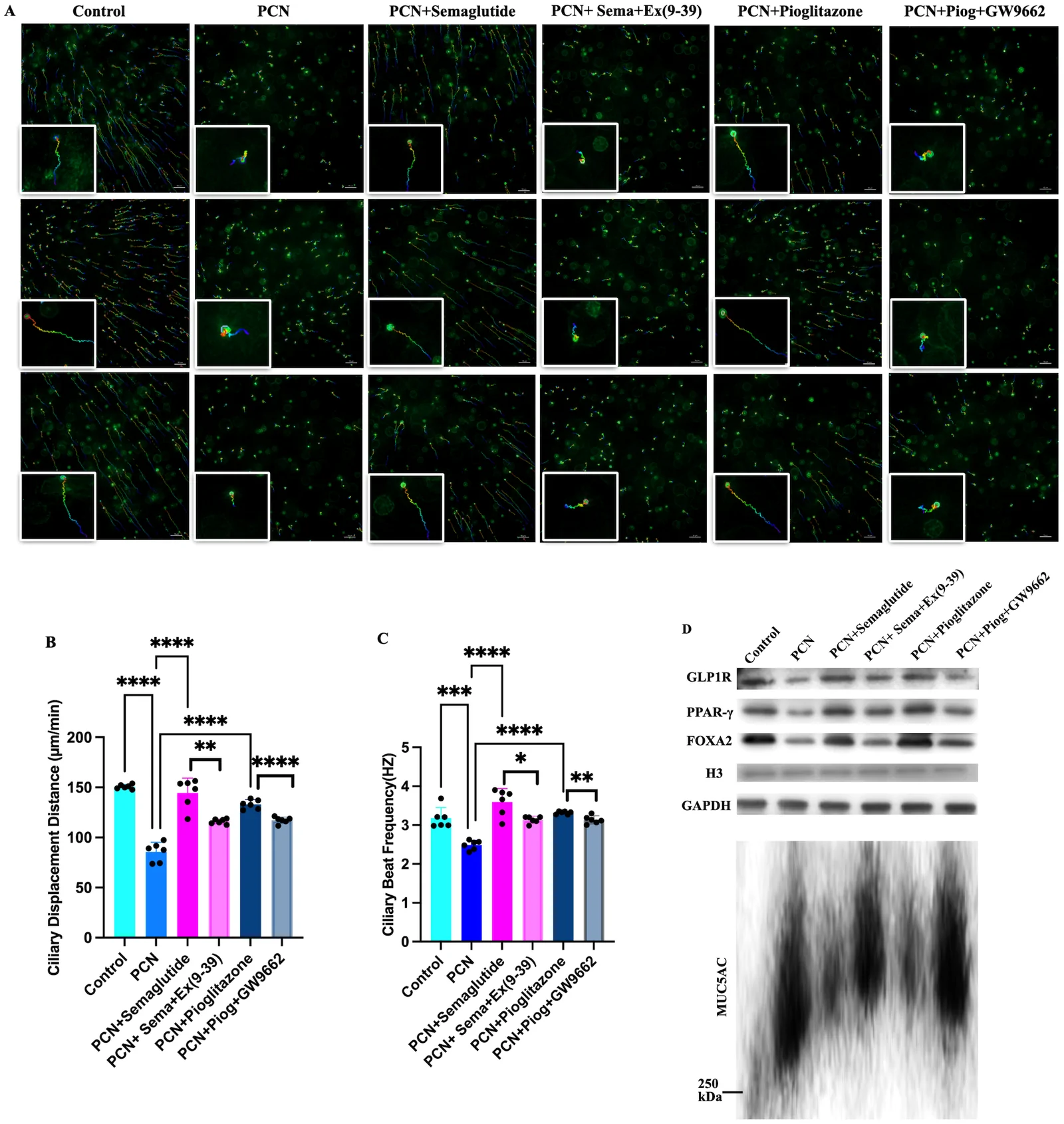
GLP1R antagonist Exendin(9-39) and PPARγ antagonist GW9662 competitively attenuate the protective effects of semaglutide and pioglitazone. The overall experimental scheme is provided in **Supplementary Figure 4**, and is similar to **Figure 5**. Briefly, ALI cultures of SAECs were pretreated with Exendin 9-39 (Ex(9-39), 200 nM) and GW9662 (20 µM) for 1 hour, followed by Semaglutide (Sema, 1 µM) and Pioglitazone (Piog, 10 µM) for 1 hour, and then with 24 hours of exposure to 5 µg/mL PCN. Then, fluorescent beads were added to the apical surface of the ALI cultures, and CDD and CBF were tracked and recorded using a Zeiss LSM 980 confocal microscope, and the data were analyzed using the Imaris software. **(A)** The tracks of the beads’ movement and their distance. The experiments were performed independently twice in triplicate. **(B, C)** Qualification of the CDD and CBF. Each dot represents one independent replicate, and each replicate has 200~400 FITC-labeled beads. **(D)** The western blot of key targets of the signaling pathway using proteins extracted from cells in **(A)**. All data are presented as mean ± s.e.m. from both experiments. *p < 0.05, **p < 0.01, ***p < 0.001, ****p < 0.0001 as analyzed by one-way ANOVA followed by Tukey’s multiple comparisons test and T-test.

To confirm that Semaglutide and Pioglitazone restore ciliary movement and mucus homeostasis through GLP1-Receptor and PPARγ, we “break the axis” by pretreating the cells with the GLP1R antagonist, Exendin(9-39), and the PPARγ antagonist, GW9662, to interfere with their respective agonists in mucociliary function assays using the ALI-cultured SAECs. We found that pretreatment with Exendin(9-39) significantly diminished Semaglutide-mediated restoration of mucociliary transport, leading to reduced CDD and CBF. Similarly, the improvements in mucus transport and ciliary function induced by Pioglitazone were significantly attenuated by GW9662 pretreatment (**Figure 6A**). Interestingly, neither antagonist completely diminished the protective effects of its respective agonist, as both CDD and CBF remained slightly improved relative to the PCN group (**Figures 6B, C**), suggesting that competitive binding to GLP1R and PPARγ, respectively, by the antagonists did not fully outcompete the binding by the agonists.

To further authenticate the underlying mechanisms, we examined the expression of key proteins by immunoblotting. The results show that pretreatment with Exendin(9–39) or GW9662 attenuated the agonist-induced restoration of GLP1R, PPARγ, and FOXA2 expression in the ALI-cultured SAECs used in the mucociliary transport measurement (**Figure 6D**). Consistent with these findings, both antagonists also derepressed MUC5AC expression. Collectively, these results indicate that Semaglutide and Pioglitazone restore the ciliary function through GLP1R-PPARγ-dependent signaling pathways.

### 3.7 Semaglutide and pioglitazone normalize FOXA2 expression, mucus homeostasis, and clearance of *P. aeruginosa* in a mouse model of chronic pyocyanin exposure

**Figure 7:**
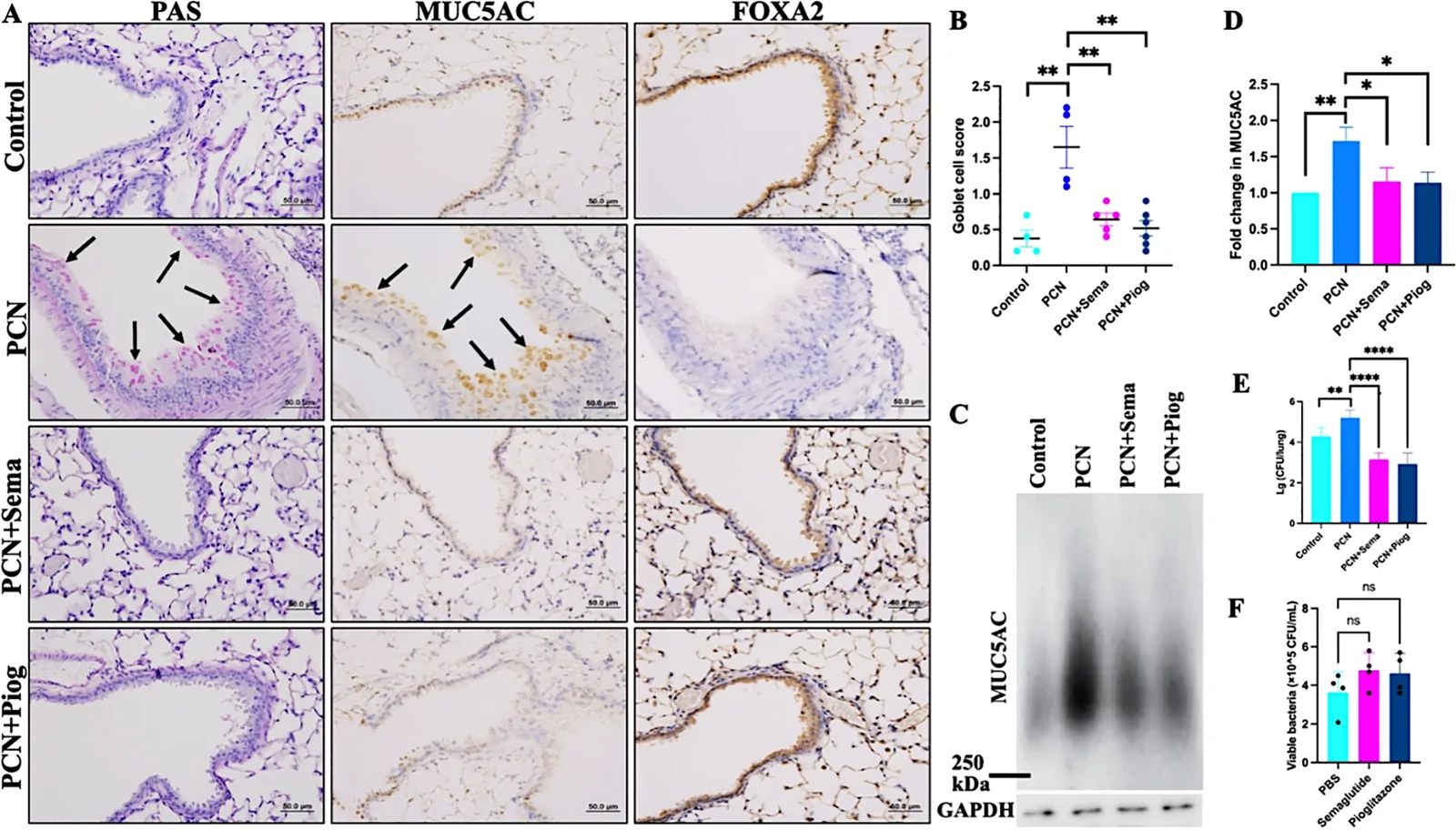
Semaglutide and pioglitazone restore FOXA2 expression and reduce mucin induction by pyocyanin (PCN) and improve *Pseudomonas aeruginosa* (PA) clearance in mouse lungs. 6-week-old C57BL6 mice (n = 8) were intranasally exposed to PCN (37.5 µg in 50 µl, once daily) and subcutaneously injected with semaglutide (160 µg/kg, daily) and pioglitazone (165 µg/kg, daily) for 2 weeks. **(A)** Mouse lungs were collected and stained using periodic acid-Schiff (PAS) and immunohistochemistry (IHC) with antibodies against FOXA2 and MUC5AC. **(B)** Goblet cell scores in PAS-stained airways. **(C)** The expression of secreted MUC5AC in the mouse bronchoalveolar lavage fluid (BALF) following PCN exposure was analyzed with agarose-acrylamide gels, with GAPDH as the loading control. Additional Western blots are shown in **Supplementary Figure 5B**. **(D)** Densitometry analysis of Western blots in C and **Supplementary Figure 5B** using ImageJ. **(E)** 6-week-old C57BL6 mice (n = 8) were exposed to PCN with or without semaglutide (Sema) or pioglitazone (Piog) for 3 weeks (25 µg in 50 µl, once daily) as described in **(A)** Then, the mice were intranasally infected with *P. aeruginosa* strain PAO1 on Days 1, 3, and 5 (3.4 x 10^6^ CFU, 2.83 x 10^6^ CFU, and 3.72 x 10^6^ CFU, respectively). On Day 7, mice were euthanized and bacterial burden determined by serial dilution plating of lung homogenates on the *Pseudomonas* isolation agar. **(F)** 5.45 x 10^5^ CFU of *P. aeruginosa* strain PAO1 were incubated in MEM and treated immediately with PBS vs. semaglutide (1 uM) vs. pioglitazone (10 uM) in 24-well plates for 3 hours in 37°C with 5% CO_2_. Viable bacterial counts were determined at the experimental endpoint by serial dilution plating on the *Pseudomonas* isolation agar. All data are presented as mean ± s.e.m. *p < 0.05, **p < 0.01, ****p < 0.0001 as analyzed by one-way ANOVA followed by Tukey’s multiple comparisons test.

Because Semaglutide and Pioglitazone significantly improve mucus homeostasis and mucociliary clearance in PCN-exposed human SAECs, we further examined their therapeutic efficacy on mucus homeostasis *in vivo.* C57BL6 mice were chronically exposed to PCN daily (25 µg in 50 µl) for three weeks, with simultaneous once-daily treatment with Semaglutide (160 µg/kg) or Pioglitazone (165 µg/kg) throughout. Negative control cohorts include unexposed mice or PCN-exposed mice subcutaneously injected with 50 µl of sterile PBS. There was a temporary weight loss for 7 days in all but PBS-treated groups, but no significant weight differences by Day 10, although the Semaglutide cohort had the lowest weight throughout the 3-week exposure (**Supplementary Figure 6A**). PAS staining revealed that when compared to lungs in the unexposed control group, there was a significant increase in the amount of glycoprotein (pink spots, black arrows) in airway-lining cells within PCN-challenged PBS-treated mouse lungs, accompanied by a significant increase in MUC5AC expression as revealed by immunohistochemical staining (**Figure 7A**). To evaluate the regulatory effects of the drugs on airway goblet cell transdifferentiation, which reflects airway remodeling, we quantified goblet cell scores from PAS-stained lung sections according to previously described criteria (39, 40). The number of goblet cells was significantly increased in PCN-challenged mouse airways but remained near basal levels under treatment of both Semaglutide and Pioglitazone during the PCN challenge (**Figures 7A, B**), suggesting that goblet cell transdifferentiation and mucus production could be attenuated by the drugs. Similarly, FOXA2 levels in airway-lining cells were diminished by PCN challenge but restored by Semaglutide and Pioglitazone (**Figure 7A**), suggesting that increased FOXA2 expression in airway-lining cells by Semaglutide and Pioglitazone attenuated *MUC5AC* gene transcription and mucus production by goblet cells. Additionally, the amounts of secreted airway mucus were significantly attenuated by Semaglutide and Pioglitazone as revealed by lowered levels of MUC5AC mucin in mouse BALFs (**Figures 7C, D**, **S6B**).

Because mucus accumulation creates a favorable microenvironment for microbial colonization, we examined whether a reduction in mucus burden by Semaglutide and Pioglitazone could limit *P. aeruginosa* colonization in PCN-challenged mouse lungs. The burden of *P. aeruginosa* strain PAO1 decreased in the Semaglutide and Pioglitazone-treated groups compared to the PCN-challenged group (**Figure 7E**). These findings suggest that Semaglutide and Pioglitazone improve mucus homeostasis and facilitate the clearance of bacteria colonizing the mucus-laden airways.

To rule out the possibility that Semaglutide or Pioglitazone have direct antibacterial activities and contribute to lower bacterial burden, PAO1 cells were incubated with vehicle, Semaglutide, or Pioglitazone in MEM, PBS, LB, or Mueller–Hinton medium, in the absence of host cells. None of the treatments significantly altered viable bacterial counts under all tested conditions (**Figure 7F**, **S6C–E**), indicating that these drugs do not exert detectable direct antibacterial effects at the concentrations examined in our studies. These findings suggest that the improved bacterial clearance observed *in vivo* is more likely mediated indirectly through restoration of mucociliary function and mucus homeostasis.

### 3.8 Semaglutide and pioglitazone selectively attenuate pro-mucin overexpression type 2 immune response *in vivo*

**Figure 8:**
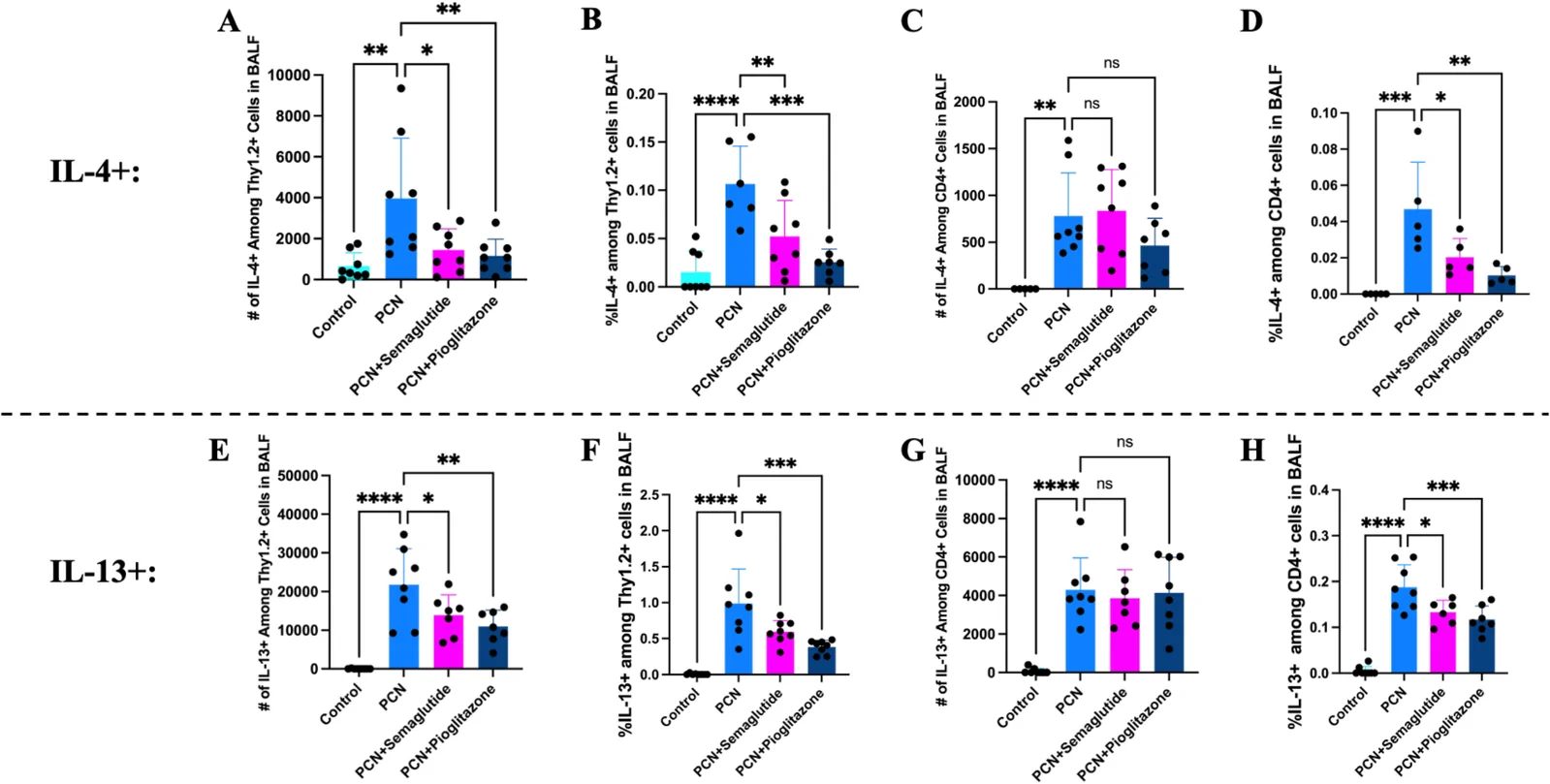
Semaglutide and pioglitazone differentially regulate pulmonary immune responses in a chronic pyocyanin-induced mouse model. C57BL/6 mice (cohorts of 8, sex-matched) were subcutaneously injected once daily with Semaglutide (160 µg/kg) and pioglitazone (165 µg/kg), followed by intranasal instillation of 37.5 µg of PCN (in 50 µl) once daily. Control cohorts were subcutaneously injected with 50 µl sterile PBS. After two weeks, mice were euthanized, and bronchoalveolar lavage fluid (BALF) was collected for immune cell analysis. **(A–D)** IL-4-producing cells in BALF, shown as the absolute number and percentage of IL-4^+^ cells among Thy1.2^+^ cells **(A, B)** and the absolute number and percentage of IL-4^+^ cells among CD4^+^ T cells **(C, D)**. **(E–H)** IL-13-producing cells in BALF, shown as the absolute number and percentage of IL-13^+^ cells among Thy1.2^+^ cells **(E, F)** and the absolute number and percentage of IL-13^+^ cells among CD4^+^ T cells **(G, H)**. All data are presented as mean ± s.e.m., and outliers were removed using GraphPad. *p < 0.05, **p < 0.01, ***p < 0.001, ****p < 0.0001 as analyzed by one-way ANOVA followed by Tukey’s multiple comparisons test.

Next, we examined whether Semaglutide and Pioglitazone also modulate inflammatory responses *in vivo*. Type 2 immune responses are associated with higher mucus production (41–43). Previously, we have shown that prolonged exposure to PCN polarized towards the type 2 response dominated by interleukin (IL)-4/IL-13-STAT6-SPDEF signaling, which is known to promote goblet cell metaplasia and mucin overproduction by suppressing FOXA2 (14, 21, 44). Because the restoration of the FOXA2 being a major focus of our study, we investigated whether Semaglutide and Pioglitazone could modulate type 2 immune responses associated with mucus hypersecretion in PCN exposure mouse models. Flow cytometry analysis revealed that PCN exposure significantly increased the numbers and the frequencies of IL-4^+^ and IL-13^+^ cells in both the Thy1.2^+^ and CD4^+^ populations in mouse lung BALF (**Figures 8A–H**), indicating enhanced type-2 immune polarization. Treatment with Semaglutide or Pioglitazone markedly attenuated type-2 responses, significantly reducing IL-4^+^ and IL-13^+^ cell populations in PCN-challenged mice. Notably, Pioglitazone exhibited a stronger suppressive effect on IL-13^+^ cells.

To further determine whether these changes reflected a broader suppression of pulmonary inflammation, we also quantified neutrophils, IFN-γ^+^, and IL-17A^+^ immune cells. Chronic PCN exposure significantly induced the infiltration of neutrophils and IFN-γ and IL-17A-producing lymphocytes (**Supplementary Figures 7A–L**). However, neither Semaglutide nor Pioglitazone substantially altered neutrophil accumulation, IFN-γ or IL-17A responses, although Pioglitazone modestly reduced the percentage of neutrophils in lung tissue. Collectively, these findings suggest that Semaglutide and Pioglitazone do not broadly suppress PCN-induced pulmonary inflammation but rather selectively attenuate the pro-mucin type 2 immune response associated with FOXA2 suppression and mucus hypersecretion.

### 3.9 FOXA2 expression is attenuated in COPD airways

**Figure 9:**
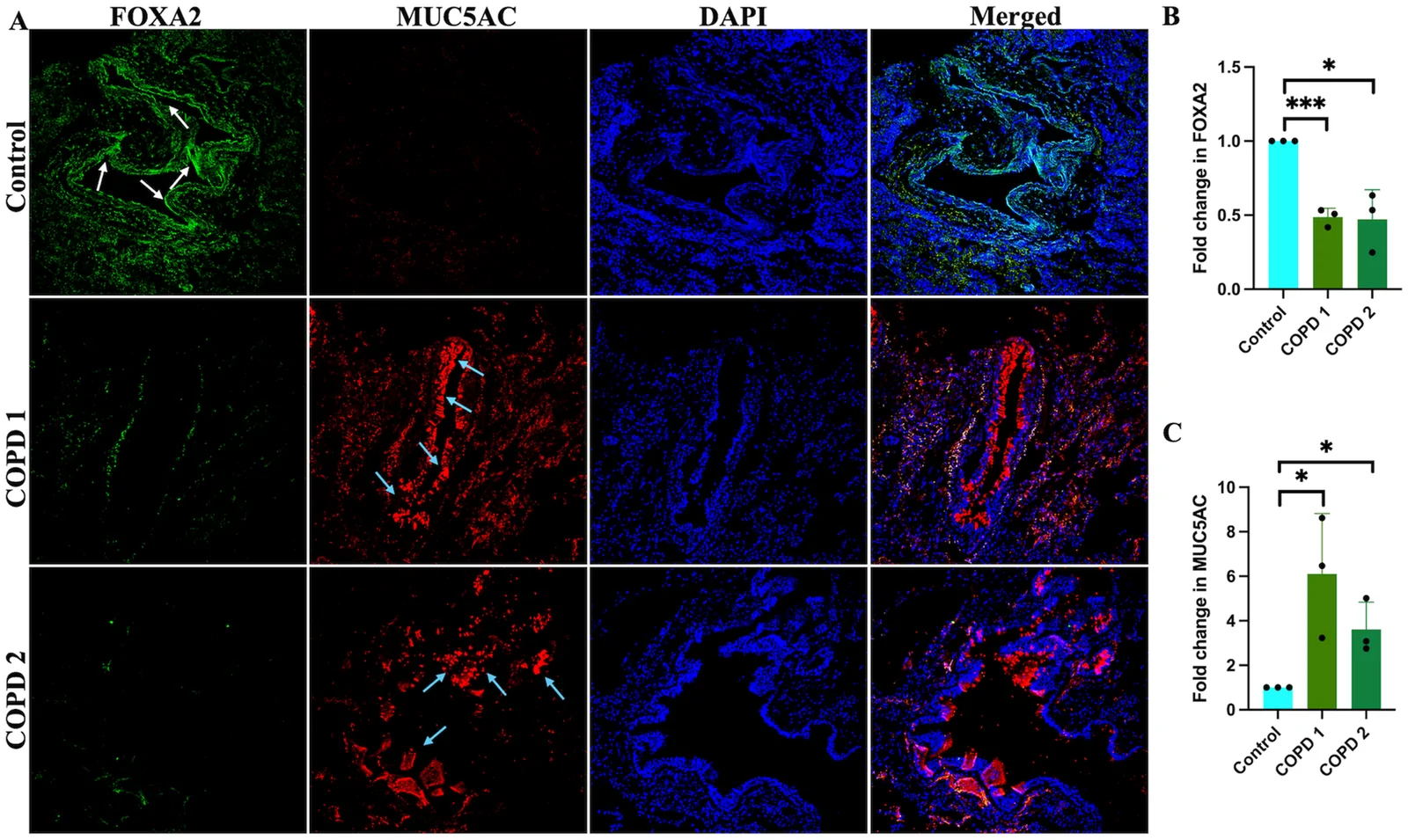
FOXA2 expression is depleted in mucus-overproducing regions of human COPD airways. Representative lung sections from de-identified control donors and patients with COPD were analyzed by immunofluorescence. Tissue sections were co-stained for FOXA2 (green) and the goblet cell–associated mucin marker MUC5AC (red), with nuclei counterstained using DAPI (blue). Merged images demonstrate the spatial relationship between FOXA2 expression and mucus-producing epithelial cells. **(A)** In control airways, FOXA2 is broadly expressed throughout the airway epithelium, with minimal MUC5AC signal. In contrast, COPD airways display robust MUC5AC expression accompanied by a marked reduction or absence of FOXA2 in mucus-producing epithelial regions. White arrows indicate epithelial cells retaining FOXA2 expression, whereas areas enriched for MUC5AC-positive cells (blue arrows) exhibit diminished FOXA2 signal. **(B, C)** Quantitative analysis of fluorescence intensity and cell-specific marker expression revealed a significant decrease in FOXA2-positive epithelial cells in COPD airways, accompanied by a corresponding increase in MUC5AC-positive goblet cells, demonstrating an inverse correlation between FOXA2 expression and mucus hypersecretion. Images were acquired at 200× magnification. Additional images from the same lung sections are shown in **Supplementary Figure 7**. All data are presented as mean ± s.e.m. *p < 0.05, **p < 0.01, ***p < 0.001, ****p < 0.0001 as analyzed by unpaired t test.

Previous studies in a handful of patients have shown that FOXA2 expression is depleted in human airways with bronchopulmonary dysplasia (13), bronchiectasis (13), asthma (45), COPD (19), and in canine COPD (20) patients. We performed immunofluorescence staining in control and two additional COPD lungs. FOXA2 expression was severely depleted in airway epithelial cells expressing abundant MUC5AC in COPD patients with stable disease at both Gold stage IV (**Figure 9**, **S8**). In contrast, airway epithelium from control lungs showed higher FOXA2 expression with minimal MUC5AC expression.

## 4 Discussion

In this study, we screened the newer generation of GLP1R and PPARγ agonists and identified Semaglutide and Pioglitazone as the best agonists in their respective classes in reducing PCN-mediated ROS production and mucus hypersecretion. Also, both agonists significantly improved the mucociliary escalator function and restored CBF and CDD in the ALI cultures of SAECs. We confirm that Semaglutide and Pioglitazone attenuate mucus hypersecretion by augmenting the GLP1R-PKA-PPARγ-PTEN/PTP1B-FOXA2 signaling axis. Activation of PTEN and PTP1B dephosphorylates and inactivate key kinases such as pEGFR, pAKT, and pERK1/2, as well as pSTAT6 within both EGFR-AKT/ERK1/2 and STAT6-SPDEF signaling cascades, thereby disrupting the transcriptional program that induces pathological goblet cell differentiation. In addition, both agonists significantly attenuated type 2 immune response, which is a major driver of goblet cell transdifferentiation and mucus hypersecretion. Restoration of FOXA2-regulated mucus homeostasis was reflected in the increased ability of mice to clear airway infection, likely mediated by reduced mucus accumulation and enhanced mucociliary clearance, as well as selective modulation of pulmonary immune responses. Because the present study was not designed to distinguish the relative contribution of these mechanisms, further investigations employing immune cell depletion, conditional knockout models, or assessment of epithelial antimicrobial peptide production will be necessary to define the precise mechanisms responsible for the enhanced bacterial clearance. Another limitation of the present study is that we assessed bacterial burden only at a single endpoint. Future studies incorporating serial bacterial quantification will be necessary to define the temporal dynamics of bacterial clearance *in vivo*.

Semaglutide (Ozempic^®^), a novel and widely used GLP1R agonist, is developed using Novo Nordisk’s proprietary protein-acylation technology (46). It lowers blood glucose levels by stimulating insulin release and decreases appetite, contributing to weight loss, as shown in **Supplementary Figure 5A**. In contrast to human GLP-1 and the first generation of GLP1 analog, Ex-4, Semaglutide has a significantly extended plasma half-life and drug potency due to specific structural modification. Semaglutide shares 94% sequence homology with GLP-1 but incorporates two amino acid substitutions at alanine at position 8 and lysine at position 34 to prevent degradation by DPP-4. Furthermore, acylation of lysine at position 26 with a hydrophilic spacer and a C18 fatty diacid chain significantly increases albumin binding, ensuring prolonged therapeutic effects *in vivo* (47). These modifications result in a long plasma half-life for Semaglutide, enabling once-weekly administration with prolonged regulatory effects despite a threefold reduction in the GLP-1 receptor affinity when compared to liraglutide (48). Semaglutide exhibits 1.4-fold greater potency in boosting cAMP levels (48). This supports our findings that Semaglutide exerts the most potent suppressive effects on PCN-induced mucin biosynthesis *in vitro*, a condition independent of metabolic degradation. Additionally, it was reported that Semaglutide protected against atherosclerosis independently of body weight, likely via its anti-inflammatory effects in mouse models (49). Not surprisingly, as we have shown in the current studies, Semaglutide outperformed Ex-4 in attenuating ROS production and in mitigating mucus overexpression and secretion in airway cells stimulated with PCN in both *in vitro* and in mouse exposure models.

Pioglitazone is a thiazolidinedione that activates the PPARγ, which governs the expression of various proteins involved in glucose and lipid metabolism. Activation of PPARγ by Pioglitzone sensitizes cellular responses to insulin by modulating the expression of genes regulating adipocyte differentiation, lipid metabolism, and glucose transport, thereby improving peripheral glucose utilization without directly increasing endogenous insulin secretion. Moreover, Pioglitazone promotes the expression of glucose transporters, such as GLUT1 and GLUT4, and enhances insulin-mediated intracellular signaling cascades, thereby contributing to improved metabolic homeostasis (50). Also, Semaglutide and Pioglitazone significantly improve metabolic disorders in T2D patients, including lowered lipid levels, reduced inflammatory responses, correction of hepatic lipid deposition, and protection of cardiovascular and renal functions (51–54). Additionally, it was reported that Semaglutide protects against atherosclerosis independently of body weight, likely via its anti-inflammatory effects in mouse models (49).

**Figure 10:**
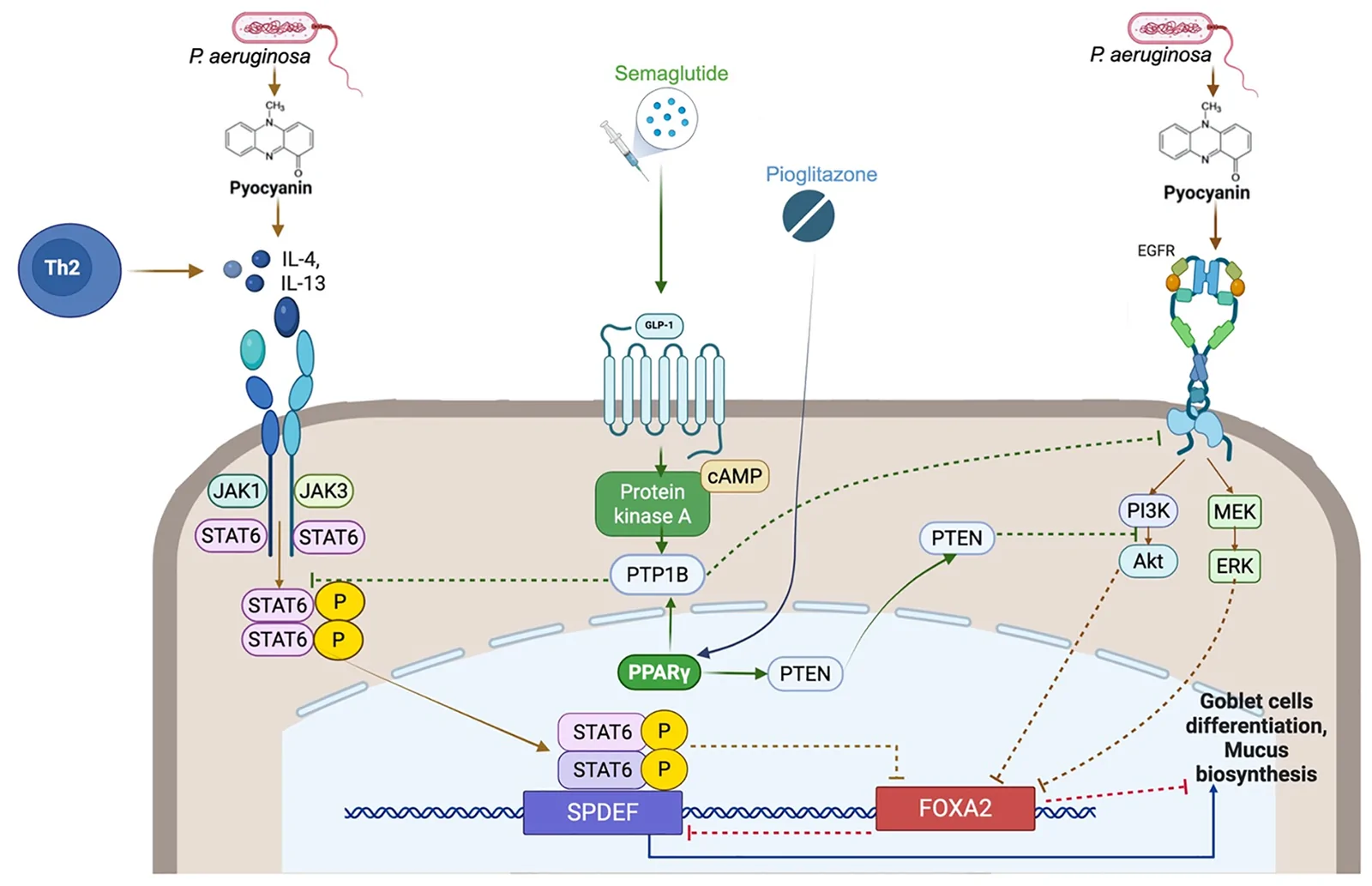
Semaglutide and pioglitazone attenuate the *Pseudomonas aeruginosa* pyocyanin-induced mucus pathology by reestablishing the GLP1R-PKA-PPARγ-FOXA2-phosphatases PTEN/PTP1B signaling.

Our previous study has shown that signal transductions induced by GLP1R and PPARγ agonists are converged in PPARγ that synergistically downregulates airway mucin biosynthesis under PCN exposure (19). PPARγ orchestrates airway mucus homeostasis by repressing transcription of mucin genes as well as modulating polarization of immune cells (19, 55). PPARγ activation enhances anti-inflammatory M2 macrophage polarization (56) and immunosuppressive phenotype in dendritic cells (57). In T cell polarization, PPARγ potentiates polarization of anti-inflammatory subsets by suppressing Th2 cytokine production (58) and enhancing regulatory T cell (Treg) differentiation (59). Given that PPARγ activation attenuates inflammatory responses, Semaglutide and Pioglitazone could resolve PCN-induced mucus hypersecretion by rewiring pulmonary immunity through reharmonizing immune cell profiling and their crosstalk. Our results presented throughout suggest that Semaglutide and Pioglitazone attenuate goblet cell mucus overexpression by inhibiting the Th2 cytokines-pSTAT6-SPDEF and pEGFR-pAKT/pERK1/2 signaling cascades (**Figure 10**). Interestingly, a recent study has suggested that the anti-inflammatory effects of Semaglutide may indirectly modulate the function of neutrophil against implant-related E. coli infection in metabolically diseased diabetic and obese mice (60), although we could only see a modest neutrophil influx modulatory effect by Pioglitazone in our PCN-exposed lungs. Another limitation of the present study is that Th2 immune responses were evaluated primarily by intracellular flow cytometric detection of IL-4- and IL-13-producing lymphocytes. Although this approach identifies cytokine-producing cells, complementary assessment of GATA3 expression and cytokine concentrations by ELISA or multiplex assays would provide additional validation and should be included in future studies.

Previously, we and others have shown that PCN-mediated oxidative stress damages mitochondrial function and depletes ATP needed for proper ciliary function (61–65). One exciting and unexpected finding from our studies is that both Semaglutide and Pioglitazone were able to restore mucociliary escalator functions by restoring the CBF and CDD inhibited by PCN (**Figures 5**, **6**). The airway mucociliary clearance (MCC), also known as the airway ciliary escalator, relies on the coordinated interaction between the mucus layer and the underlying motile cilia that line the airway epithelium, in which inhaled particles are entrapped in mucus and subsequently cleared toward the oropharynx through directional ciliary beating (66). The efficiency of the MCC system depends on three key factors: CBF, the quantity and properties of airway secretions, and periciliary fluid depth, which is regulated by ion transport (67). It was reported that protein kinase C (PKC) produces CBF enhancement via activation of Ca^2+^ influx through non-voltage-operated Ca^2+^ channels. This Ca^2+^ influx seems to be responsible for the duration of ciliary stimulation produced by the extracellular ATP (68). In the airways, CBF is stimulated via three second messengers: Ca^2+^, cGMP, and cAMP, all of which could increase CBF via distinct pathways. Ca^2+^ is released from intracellular storage by IP3, activated by P2Y2 through ATP, and by muscarinic receptors by acetylcholine (ACh). Mechano-stimulation and high viscosity increase intracellular Ca^2+^ by ATP release through pannexin with stimulation of P2Y2 and through activation of TRPV4. Released Ca^2+^ binds to CaM and leads to increased production of cAMP and cGMP, which increases CBF through PKA and PKG by phosphorylation of dynein cytoskeletal motor proteins that drive ciliary motility. Specifically, ACh, ATP, and cAMP stimulation increase CBF (69). In addition, it was found that phosphodiesterase isoenzyme 1A (PDE1A) inhibition induced by low intracellular Ca²^+^ enhances CBF due to cAMP accumulation (70). We are actively investigating whether Semaglutide and Pioglitazone modulate the aforementioned signaling network to restore MCC.

While classical PKC isoforms like PKCα promote CBF through Ca²^+^-dependent mechanisms, protein kinase C epsilon (PKCϵ) appears to play an inhibitory role independent of Ca²^+^, possibly as a negative feedback or stress response mechanism. More recent studies have shown that agents that slow cilia beating (organic dusts, alcohol and aldehydes, bisulfites, neuropeptides, microbial pathogens, cigarette smoke, etc.) share a common theme: PKCϵ activation (71–74). PKCϵ is a PKC isotype characterized as a Ca^2+^-independent, phorbol ester/diacylglycerol-sensitive serine/threonine kinase (75), which leads to airway ciliary slowing, and its auto-downregulation causes ciliated cell detachment (76). Furthermore, the closing connexin/pannexin hemichannels that release ATP and decrease intracellular Ca²^+^ levels may also suppress the CBF (77). Although the detailed mechanisms underlying the negative regulation of CBF by PKCϵ remain poorly understood, targeting PKCϵ may offer therapeutic potential for diseases characterized by impaired mucociliary clearance. We are actively investigating whether PCN modulates the PKCϵ signaling, which is beyond the scope of current studies and will be reported elsewhere in the future. In summary, our study identifies a promising adjunctive therapy for COPD and chronic airway diseases with impaired ciliary function by repurposing FDA-approved Semaglutide and Pioglitazone.

## Data Availability

The original contributions presented in the study are included in the article/**Supplementary Material**. Further inquiries can be directed to the corresponding author.

## References

[1] Li Y Li J Yang H Zhang Y . Effect of triple therapy on mortality and cardiovascular risk in patients with moderate to severe COPD: a meta-analysis of randomized controlled trials. BMC Pulm Med. (2025) 25:345. doi: 10.1186/s12890-025-03823-6 40684148 PMC12276660

[2] Chow R Pham M Cope M Wang J Ghasemi R Yang S . Sexually dimorphic response to tobacco exposure in COPD: a systematic review and meta-analysis. BMC Pulm Med. (2026) 26:45. doi: 10.1186/s12890-025-04079-w 41492127 PMC12870387

[3] Sood A Petersen H Blanchette CM Meek P Picchi MA Belinsky SA . Wood smoke exposure and gene promoter methylation are associated with increased risk for COPD in smokers. Am J Respir Crit Care Med. (2010) 182:1098–104. doi: 10.1164/rccm.201002-0222oc 20595226 PMC3001253

[4] Dandurand RJ Bourbeau J Eidelman DH . Impact of Fev1 and acute exacerbation rate (aer) on the Gold 2011 grading of COPD in a community based respirology practice. Am J Respir Crit Care Med. (2013) 187:A3938.

[5] Lau GW Hassett DJ Ran H Kong F . The role of pyocyanin in Pseudomonas aeruginosa infection. Trends Mol Med. (2004) 10:599–606. doi: 10.1016/j.molmed.2004.10.002 15567330

[6] Lew SQ Chong SY Lau GW . Modulation of pulmonary immune functions by the Pseudomonas aeruginosa secondary metabolite pyocyanin. Front Immunol. (2025) 16:1550724. doi: 10.3389/fimmu.2025.1550724 40196115 PMC11973339

[7] Kuo SH Lau GW . Pseudomonas aeruginosa-derived metabolites and volatile organic compounds: impact on lung epithelial homeostasis and mucosal immune response. Front Immunol. (2025) 16:1553013. doi: 10.3389/fimmu.2025.1553013 40552284 PMC12183278

[8] Yang Y Wang K Chen S . Effects of hypoglycemic agents on pulmonary diseases: a comprehensive narrative review. J Inflammation Res. (2025) 18:18053–78. doi: 10.2147/jir.s556790 41497535 PMC12765701

[9] Joskova M Sadlonova V Mokra D Kocan I Sutovska M Kackova K . Respiratory ciliary beat frequency in COPD: balancing oxidative stress and pharmacological treatment. Antioxidants (Basel). (2025) 14:1340. doi: 10.3390/antiox14111340 41300497 PMC12649418

[10] Ganesan S Comstock AT Sajjan US . Barrier function of airway tract epithelium. Tissue Barriers. (2013) 1:e24997. doi: 10.4161/tisb.24997 24665407 PMC3783221

[11] Rose MC Voynow JA . Respiratory tract mucin genes and mucin glycoproteins in health and disease. Physiol Rev. (2006) 86:245–78. doi: 10.1152/physrev.00010.2005 16371599

[12] Fahy JV Dickey BF . Airway mucus function and dysfunction. N Engl J Med. (2010) 363:2233–47. doi: 10.1056/nejmra0910061 21121836 PMC4048736

[13] Wan H Kaestner KH Ang SL Ikegami M Finkelman FD Stahlman MT . Foxa2 regulates alveolarization and goblet cell hyperplasia. Development. (2004) 131:953–64. doi: 10.1242/dev.00966 14757645

[14] Choi W Choe S Lau GW . Inactivation of FOXA2 by respiratory bacterial pathogens and dysregulation of pulmonary mucus homeostasis. Front Immunol. (2020) 11:515. doi: 10.3389/fimmu.2020.00515 32269574 PMC7109298

[15] Kohri K Ueki IF Shim JJ Burgel PR Oh YM Tam DC . Pseudomonas aeruginosa induces MUC5AC production via epidermal growth factor receptor. Eur Respir J. (2002) 20:1263–70. doi: 10.1183/09031936.02.00001402 12449183

[16] Chen G Korfhagen TR Xu Y Kitzmiller J Wert SE Maeda Y . SPDEF is required for mouse pulmonary goblet cell differentiation and regulates a network of genes associated with mucus production. J Clin Invest. (2009) 119:2914–24. doi: 10.1172/jci39731 19759516 PMC2752084

[17] Kesimer M Ehre C Burns KA Davis CW Sheehan JK Pickles RJ . Molecular organization of the mucins and glycocalyx underlying mucus transport over mucosal surfaces of the airways. Mucosal Immunol. (2013) 6:379–92. doi: 10.1038/mi.2012.81 22929560 PMC3637662

[18] Hao YH Kuang Z Xu Y Walling BE Lau GW . Pyocyanin-induced mucin production is associated with redox modification of FOXA2. Respir Res. (2013) 14:82. doi: 10.1186/1465-9921-14-82 23915402 PMC3765780

[19] Choi W Choe S Lin J Borchers MT Kosmidler B Vassallo R . Exendin-4 restores airway mucus homeostasis through the GLP1R-PKA-PPARgamma-FOXA2-phosphatase signaling. Mucosal Immunol. (2020) 13:637–51. doi: 10.1038/s41385-020-0262-1 32034274 PMC7664156

[20] Choi W Yang AX Waltenburg MA Choe S Steiner M Radwan A . FOXA2 depletion leads to mucus hypersecretion in canine airways with respiratory diseases. Cell Microbiol. (2019) 21:e12957. doi: 10.1111/cmi.12957 30221439

[21] Hao Y Kuang Z Walling BE Bhatia S Sivaguru M Chen Y . Pseudomonas aeruginosa pyocyanin causes airway goblet cell hyperplasia and metaplasia and mucus hypersecretion by inactivating the transcriptional factor FoxA2. Cell Microbiol. (2012) 14:401–15. doi: 10.1111/j.1462-5822.2011.01727.x 22103442

[22] Ho TW Huang CT Ruan SY Tsai YJ Lai F Yu CJ . Diabetes mellitus in patients with chronic obstructive pulmonary disease-the impact on mortality. PloS One. (2017) 12:e0175794. doi: 10.1371/journal.pone.0175794 28410410 PMC5391945

[23] Gayle A Dickinson S Poole C Pang M Fauconnot O Quint JK . Incidence of type II diabetes in chronic obstructive pulmonary disease: a nested case-control study. NPJ Prim Care Respir Med. (2019) 29:28. doi: 10.1038/s41533-019-0138-6 31308364 PMC6629671

[24] Castañ-Abad MT Montserrat-Capdevila J Godoy P Marsal JR Ortega M Alsedà M . Diabetes as a risk factor for severe exacerbation and death in patients with COPD: a prospective cohort study. Eur J Public Health. (2020) 30:822–7. doi: 10.1093/eurpub/ckz219 31951259

[25] Dogruel H Balci MK . Development of therapeutic options on type 2 diabetes in years: Glucagon-like peptide-1 receptor agonist's role intreatment; from the past to future. World J Diabetes. (2019) 10:446–53. doi: 10.4239/wjd.v10.i8.446 31523380 PMC6715574

[26] Trujillo JM Nuffer W . GLP-1 receptor agonists for type 2 diabetes mellitus: recent developments and emerging agents. Pharmacotherapy. (2014) 34:1174–86. doi: 10.1002/phar.1507 25382096

[27] Gupta O Pradhan T Chawla G . Structural and functional insights into PPAR-γ: review of its potential and drug design innovations for the development of antidiabetic agents. Eur J Med Chem. (2026) 302:118264. doi: 10.1016/j.ejmech.2025.118264 41124882

[28] Richardson H Campbell SC Smith SA Macfarlane WM . Effects of rosiglitazone and metformin on pancreatic beta cell gene expression. Diabetologia. (2006) 49:685–96. doi: 10.1007/s00125-006-0155-1 16489446

[29] Lee SY Kang EJ Hur GY Jung KH Jung HC Lee SY . Peroxisome proliferator-activated receptor-gamma inhibits cigarette smoke solution-induced mucin production in human airway epithelial (NCI-H292) cells. Am J Physiol Lung Cell Mol Physiol. (2006) 291:L84–90. doi: 10.1152/ajplung.00388.2005 16443643

[30] Lin ZZ Li ZX Jia Q . Glucagon-like peptide-1 receptor agonists in the prevention of ischemic stroke: therapeutic potential and mechanisms. J Stroke. (2025) 27:289–301. doi: 10.5853/jos.2025.00584 41084286 PMC12527581

[31] Li L Lui DTW Fong CHY Chow WS Au ICH Xiong X . Benefits of glucagon-like peptide-1 receptor agonists versus pioglitazone for cardio-hepatic outcomes: a territory-wide target trial emulation. Cardiovasc Diabetol. (2025) 24:416. doi: 10.1186/s12933-025-02973-5 41174643 PMC12577280

[32] Platz E Peikert A Bozkurt B Van der Schueren B Butler J Lingvay I . Designing next-generation cardiometabolic outcome trials for obesity medicines. J Am Coll Cardiol. (2026) 87:1936–49. doi: 10.1016/j.jacc.2026.01.013 41811272

[33] Zhou J Pineyro MA Wang X Doyle ME Egan JM . Exendin-4 differentiation of a human pancreatic duct cell line into endocrine cells:: involvement of PDX-1 and HNF3β transcription factors. J Cell Physiol. (2002) 192:304–14. doi: 10.1002/jcp.10143 12124776

[34] Ghannam IAY Abdel-Mohsen HT Ali IH . Recent advances in the design, development and therapeutic applications of PPARγ agonists. Future Med Chem. (2025) 17:2751–70. doi: 10.1080/17568919.2025.2571022 41118140 PMC12707219

[35] Wallach JD Wang K Zhang AD Cheng D Grossetta Nardini HK Lin H . Updating insights into rosiglitazone and cardiovascular risk through shared data: individual patient and summary level meta-analyses. Bmj-British Med J. (2020) 368:17078. doi: 10.1136/bmj.l7078 32024657 PMC7190063

[36] Riccioni G Notarangelo C Riccioni M D’Orazio N . Cardiovascular disease and diabetes: a new challenge in the treatment and management. Int J Mol Sci. (2025) 27:354. doi: 10.3390/ijms27010354 41516231 PMC12785618

[37] Choi W Yang AX Sieve A Kuo SH Mudalagiriyappa S Vieson M . Pulmonary mycosis drives Forkhead box protein A2 degradation and mucus hypersecretion through activation of the spleen tyrosine kinase-epidermal growth factor receptor-AKT/extracellular signal-regulated kinase 1/2 signaling. Am J Pathol. (2021) 191:108–30. doi: 10.1016/j.ajpath.2020.09.013 33069717 PMC7786105

[38] Olm MA Kögler JE Macchione M Shoemark A Saldiva PHN Rodrigues JC . Primary ciliary dyskinesia: evaluation using cilia beat frequency assessment via spectral analysis of digital microscopy images. J Appl Physiol (1985). (2011) 111:295–302. doi: 10.1152/japplphysiol.00629.2010 21551013 PMC3137538

[39] Grundstrom J Saarne T Kemi C Gregory JA Wadén K Pils MC . Development of a mouse model for chronic cat allergen-induced asthma. Int Arch Allergy Immunol. (2014) 165:195–205. doi: 10.1159/000369066 25531229

[40] Prangtaworn P Chaisri U Seesuay W Mahasongkram K Onlamoon N Reamtong O . Tregitope-linked refined allergen vaccines for immunotherapy in cockroach allergy. Sci Rep. (2018) 8:15480. doi: 10.1038/s41598-018-33680-9 30341299 PMC6195530

[41] Cohn L Homer RJ Marinov A Rankin J Bottomly K . Induction of airway mucus production by T helper 2 (Th2) cells: a critical role for interleukin 4 in cell recruitment but not mucus production. J Exp Med. (1997) 186:1737–47. doi: 10.1084/jem.186.10.1737 9362533 PMC2199146

[42] Steele H Sachen K McKnight AJ Soloff R Herro R . Targeting TL1A/DR3 signaling offers a therapeutic advantage to neutralizing IL13/IL4Rα in muco-secretory fibrotic disorders. Front Immunol. (2021) 12. doi: 10.3389/fimmu.2021.692127 34305924 PMC8299868

[43] Akdis CA Arkwright PD Brüggen MC Busse W Gadina M Guttman-Yassky E . Type 2 immunity in the skin and lungs. Allergy. (2020) 75:1582–605. doi: 10.1111/all.14318 32319104

[44] Caldwell CC Chen Y Goetzmann HS Hao Y Borchers MT Hassett DJ . Pseudomonas aeruginosa exotoxin pyocyanin causes cystic fibrosis airway pathogenesis. Am J Pathol. (2009) 175:2473–88. doi: 10.2353/ajpath.2009.090166 19893030 PMC2789600

[45] Park SW Verhaeghe C Nguyenvu LT Barbeau R Eisley CJ Nakagami Y . Distinct roles of FOXA2 and FOXA3 in allergic airway disease and asthma. Am J Respir Crit Care Med. (2009) 180:603–10. doi: 10.1164/rccm.200811-1768oc 19628779 PMC2753788

[46] Dhillon S . Semaglutide: first global approval. Drugs. (2018) 78:275–84. doi: 10.1007/s40265-018-0871-0 29363040

[47] Knudsen LB Lau J . The discovery and development of liraglutide and semaglutide. Front Endocrinol (Lausanne). (2019) 10:155. doi: 10.3389/fendo.2019.00155 31031702 PMC6474072

[48] Lau J Bloch P Schäffer L Pettersson I Spetzler J Kofoed J . Discovery of the once-weekly glucagon-like peptide-1 (GLP-1) analogue semaglutide. J Med Chem. (2015) 58:7370–80. doi: 10.1021/acs.jmedchem.5b00726 26308095

[49] Rakipovski G Rolin B Kirk R . Long-acting GLP-1 receptor agonists semaglutide and liraglutide protect against development of atherosclerosis in animal models independent of body-weight-lowering effects. Diabetes. (2017) 66:A64–4.

[50] Gillies PS Dunn CJ . Pioglitazone. Drugs. (2000) 60:333–43. doi: 10.2165/00003495-200060020-00009 10983737. discussion 344-5.

[51] Murase K Odaka H Suzuki M Tayuki N Ikeda H . Pioglitazone time-dependently reduces tumour necrosis factor-alpha level in muscle and improves metabolic abnormalities in Wistar fatty rats. Diabetologia. (1998) 41:257–64. doi: 10.1007/s001250050901 9541164

[52] Buchanan TA Meehan WP Jeng YY Yang D Chan TM Nadler JL . Blood pressure lowering by pioglitazone. Evidence for a direct vascular effect. J Clin Invest. (1995) 96:354–60. doi: 10.1172/jci118041 7615805 PMC185207

[53] Mathisen A Rubin C . The effect of pioglitazone on glucose control and lipid profile in patients with type 2 diabetes. Diabetologia. (1999) 42:A227–7. doi: 10.1016/s0168-8227(00)81657-5

[54] Li WX Wang Y Liu C Yu Y Xu L Dong B . Comparing efficacy of Chiglitazar, Pioglitazone, and Semaglutide in type 2 diabetes: A retrospective study. Diabetes Ther. (2025) 16:993–1017. doi: 10.1007/s13300-025-01724-9 40126828 PMC12006573

[55] Lakshmi SP Reddy AT Banno A Reddy RC . Airway epithelial cell peroxisome proliferator-activated receptor gamma regulates inflammation and mucin expression in allergic airway disease. J Immunol. (2018) 201:1775–83. doi: 10.4049/jimmunol.1800649 30061200 PMC6145144

[56] Bouhlel MA Derudas B Rigamonti E Dièvart R Brozek J Haulon S . PPARγ activation primes human monocytes into alternative M2 macrophages with anti-inflammatory properties. Cell Metab. (2007) 6:137–43. doi: 10.1016/j.cmet.2007.06.010 17681149

[57] Tuna H Avdiushko RG Sindhava VJ Wedlund L Kaetzel CS Kaplan AM . Regulation of the mucosal phenotype in dendritic cells by PPARγ: Role of tissue microenvironment. J Leukocyte Biol. (2014) 95:471–85. doi: 10.1189/jlb.0713408 24295831 PMC3923081

[58] Banno A Reddy AT Lakshmi SP Reddy RC . PPARs: Key regulators of airway inflammation and potential therapeutic targets in asthma. Nucl Receptor Res. (2018) 5:101306. doi: 10.11131/2018/101306 29450204 PMC5810409

[59] Cipolletta D Feuerer M Li A Kamei N Lee J Shoelson SE . PPAR-γ is a major driver of the accumulation and phenotype of adipose tissue T cells. Nature. (2012) 486:549–U151. doi: 10.1038/nature11132 22722857 PMC3387339

[60] Liu T Zhou L Chen Y Lin J Zhu H . Semaglutide outperforms insulin in restoring neutrophil function against implant-related infection in diabetic and obese mice: Experimental research. Int J Surg. (2025) 111:273–82. doi: 10.1097/js9.0000000000001896 38935106 PMC11745711

[61] Wilson R Pitt T Taylor G Watson D MacDermot J Sykes D . Pyocyanin and 1-hydroxyphenazine produced by Pseudomonas aeruginosa inhibit the beating of human respiratory cilia *in vitro*. J Clin Invest. (1987) 79:221–9. doi: 10.1172/jci112787 3098783 PMC424027

[62] Kanthakumar K Taylor G Tsang KW Cundell DR Rutman A Smith S . Mechanisms of action of Pseudomonas aeruginosa pyocyanin on human ciliary beat *in vitro*. Infect Immun. (1993) 61:2848–53. doi: 10.1128/iai.61.7.2848-2853.1993 8390405 PMC280930

[63] Murphy MP . How mitochondria produce reactive oxygen species. Biochem J. (2009) 417:1–13. doi: 10.1042/bj20081386 19061483 PMC2605959

[64] Ran H Hassett DJ Lau GW . Human targets of Pseudomonas aeruginosa pyocyanin. Proc Natl Acad Sci USA. (2003) 100:14315–20. doi: 10.1073/pnas.2332354100 PMC283589 14605211

[65] Kong F Young L Chen Y Ran H Meyers M Joseph P . Pseudomonas aeruginosa pyocyanin inactivates lung epithelial vacuolar ATPase-dependent cystic fibrosis transmembrane conductance regulator expression and localization. Cell Microbiol. (2006) 8:1121–33. doi: 10.1111/j.1462-5822.2006.00696.x 16819965

[66] Whitsett JA . Airway epithelial differentiation and mucociliary clearance. Ann Am Thorac Soc. (2018) 15:S143–8. doi: 10.1513/annalsats.201802-128aw 30431340 PMC6322033

[67] Stannard W O'Callaghan C . Ciliary function and the role of cilia in clearance. J Aerosol Medicine-Deposition Clearance Effects Lung. (2006) 19:110–5. doi: 10.1089/jam.2006.19.110 16551222

[68] Levin R Braiman A Priel Z . Protein kinase C induced calcium influx and sustained enhancement of ciliary beating by extracellular ATP. Cell Calcium. (1997) 21:103–13. doi: 10.1016/s0143-4160(97)90034-8 9132293

[69] Schmid A Salathe M . Ciliary beat co-ordination by calcium. Biol Cell. (2011) 103:159–69. doi: 10.1042/bc20100120 21401526

[70] Kogiso H Hosogi S Ikeuchi Y Tanaka S Inui T Marunaka Y . Ca(2+) ](i) modulation of cAMP-stimulated ciliary beat frequency via PDE1 in airway ciliary cells of mice. Exp Physiol. (2018) 103:381–90. doi: 10.1113/EP086681 29282782

[71] Wyatt TA Sisson JH Allen-Gipson DS McCaskill ML Boten JA DeVasure JM . Co-exposure to cigarette smoke and alcohol decreases airway epithelial cell cilia beating in a protein kinase Cϵ-dependent manner. Am J Pathol. (2012) 181:431–40. doi: 10.1016/j.ajpath.2012.04.022 22677421 PMC3409441

[72] Wyatt TA Bailey KL Simet SM Warren KJ Sweeter JM DeVasure JM . Alcohol potentiates RSV-mediated injury to ciliated airway epithelium. Alcohol. (2019) 80:17–24. doi: 10.1016/j.alcohol.2018.07.010 31235345 PMC7100607

[73] Bauer CD Mosley DD Samuelson DR Poole JA Smith DR Knoell DL . Zinc protects against swine barn dust-induced cilia slowing. Biomolecules. (2024) 14:843. doi: 10.3390/biom14070843 PMC11274422 39062557

[74] Bailey KL LeVan TD Yanov DA Pavlik JA DeVasure JM Sisson JH . Non-typeable Haemophilus influenzae decreases cilia beating via protein kinase Cepsilon. Respir Res. (2012) 13:49. doi: 10.1186/1465-9921-13-49 22712879 PMC3487807

[75] Akita Y . Protein kinase C-ϵ (PKC-ϵ):: Its unique structure and function. J Biochem. (2002) 132:847–52. doi: 10.1093/oxfordjournals.jbchem.a003296 12473185

[76] Slager RE Sisson JH Pavlik JA Johnson JK Nicolarsen JR Jerrells TR . Inhibition of protein kinase C epsilon causes ciliated bovine bronchial cell detachment. Exp Lung Res. (2006) 32:349–62. doi: 10.1080/01902140600959630 17090476 PMC2100410

[77] Zhou KQ Green CR Bennet L Gunn AJ Davidson JO . The role of connexin and pannexin channels in perinatal brain injury and inflammation. Front Physiol. (2019) 10:141. doi: 10.3389/fphys.2019.00141 30873043 PMC6400979

