# Supplementary material for: Semaglutide and pioglitazone restore airway mucosal homeostasis by resolving Pseudomonas aeruginosa pyocyanin-induced mucus hypersecretion, ciliostasis, and type 2 airway inflammation
Source: Front Immunol. 2026 Sep 7;17:1877061. doi: 10.3389/fimmu.2026.1877061 (PMC13590329; doi:10.3389/fimmu.2026.1877061)
Supplement: Supplementary file 1 [file DataSheet1.pdf]

Semaglutide and Pioglitazone restore airway mucosal homeostasis by resolving *Pseudomonas aeruginosa* pyocyanin-induced mucus hypersecretion, ciliostasis, and type 2 airway inflammation

Cong Wu[1], Nitish A. Kulkarni[1], Shi Qian Lew[1], Woosuk Choi[1], Mayandi Sivaguru[2,3,4], Beata Kosmider[5], Som G. Nanjappa[1], Gee W. Lau[1*]

[1]Department of Pathobiology, University of Illinois Urbana-Champaign

[2]Cytometry and Microscopy to Omics (CMtO) Facility, Biotechnology Center, University of Illinois Urbana-Champaign

[3]Cytometry and Microscopy to Omics Facility, Roy J. Carver Biotechnology Center, University of Illinois Urbana-Champaign

[4]Earth Science and Environmental Change, School of Earth, Society and the Environment, University of Illinois Urbana-Champaign

[5]Center for Inflammation and Lung Research, Department of Microbiology, Immunology and Inflammation, Lewis Katz School of Medicine, Temple University

***Correspondence:**
Gee W. Lau
geelau@illinois.edu

Supplementary Figures and Legends

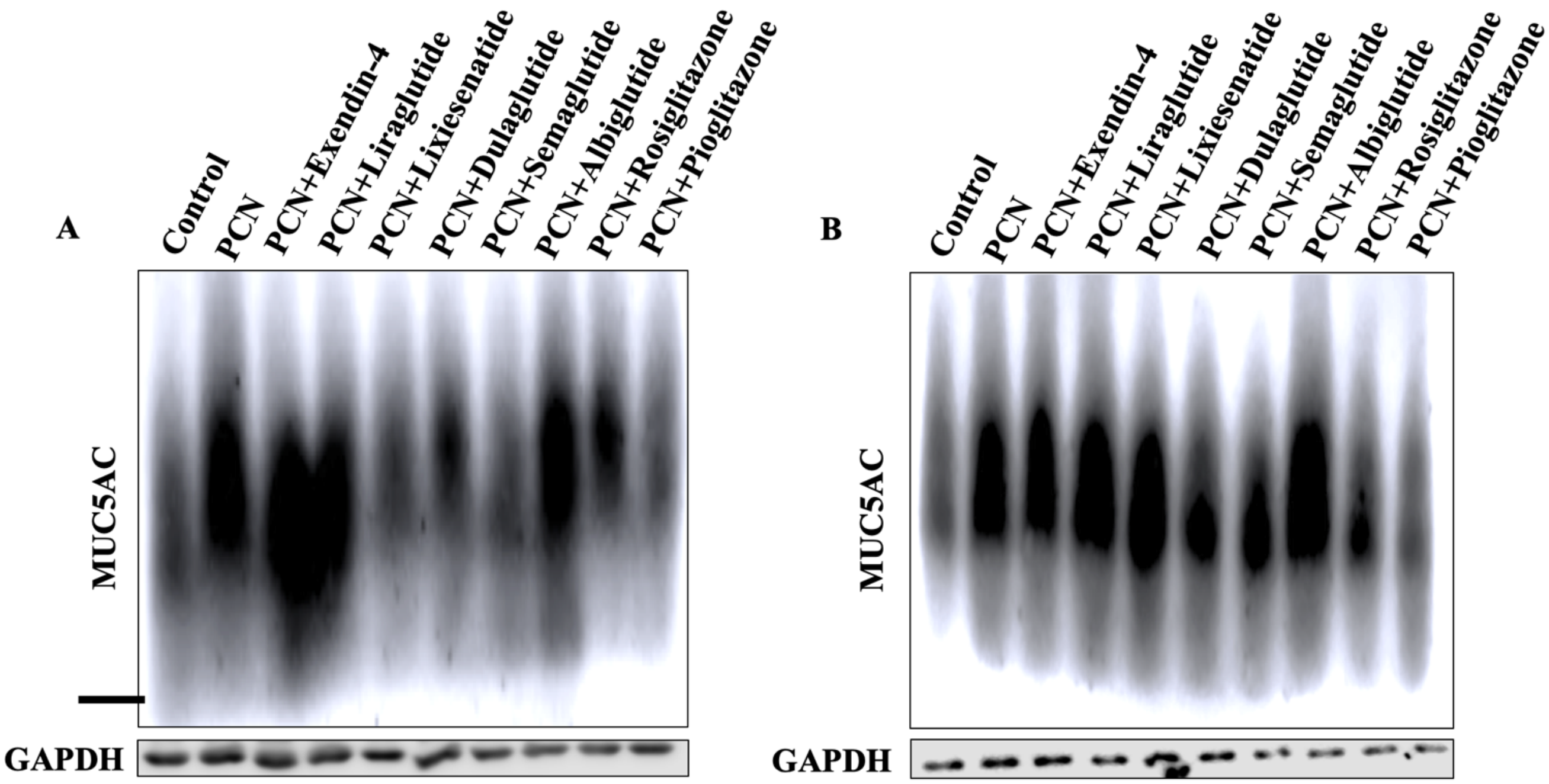

Figure S1. Semaglutide and Pioglitazone are most effective in suppressing mucin production induced by PCN in submerged 16HBEo- cells. Confluent submerged 16HBEo- cells were pretreated with each of the 6 GLP1R agonists (1 µM) and 2 PPARγ agonists (10 µM), respectively, for 1 hour before exposure to PCN (5 µg/mL) for 24 hours. **(A, B)** Western Blot analysis for all 8 agonists' efficacy to attenuate the expression of MUC5AC mucin induced by PCN. The experiments were performed independently three times. Additional MUC5AC western blot and the cumulative densitometric quantification of all three Western blots can be found in Figure 2.

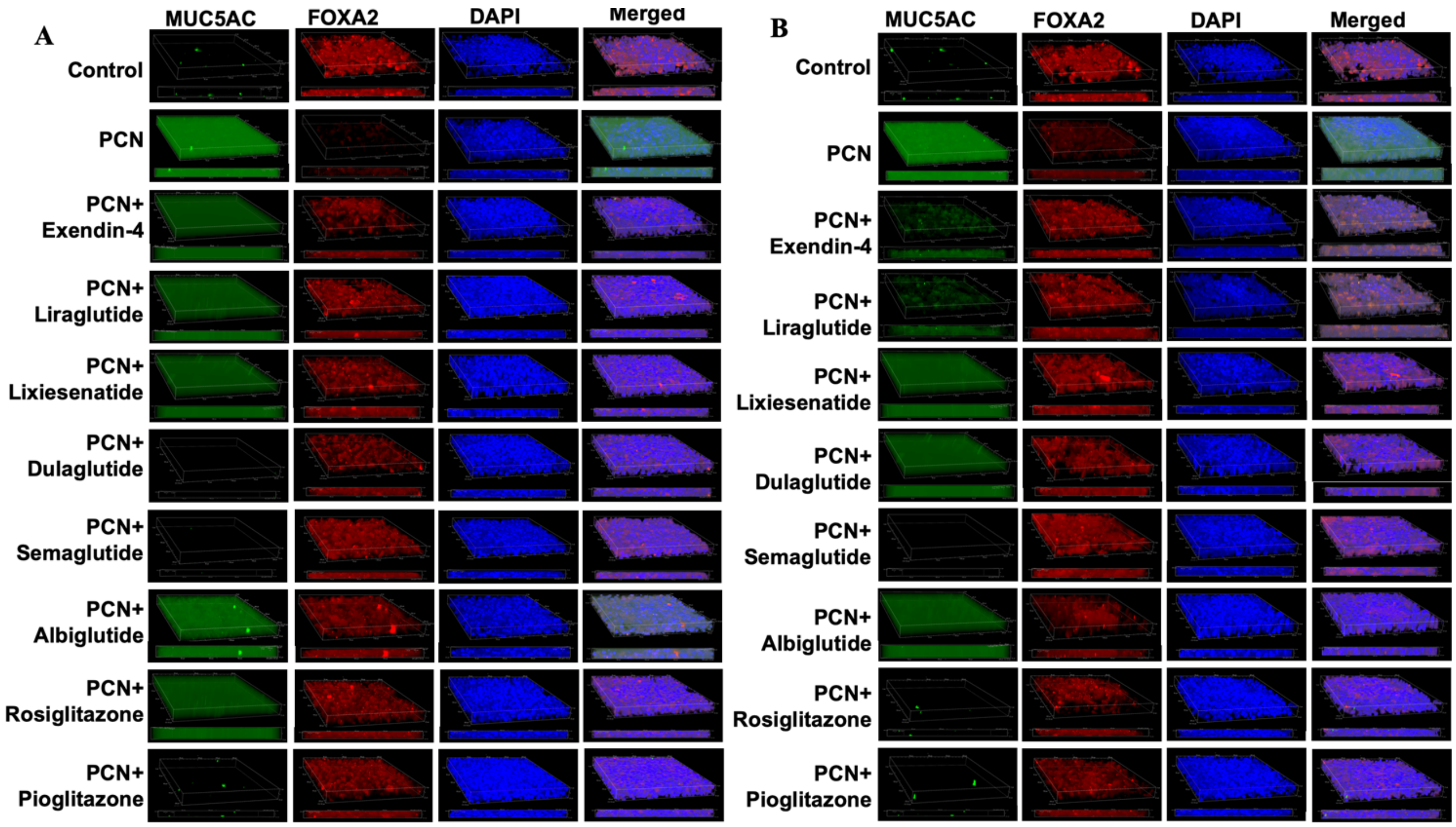

Figure S2. Semaglutide and Pioglitazone have the best efficacy in restoring FOXA2 expression and in attenuating MUC5AC mucin expression in the air-liquid interface (ALI) culture of 16HBEo- cells exposed to pyocyanin. Partially differentiated ALI cultured 16HBEo- cells (28 days after air lift) were pretreated for 1 hour with individual GLP1R agonists (1 μM) or PPARγ agonists (10 μM), followed by potentiation by PCN (5 μg/mL) for 24 hours. **(A, B)** Images of ALI cultures stained with primary antibodies and visualized with Alexa Fluor 488 (green) or Alexa Fluor 594 (red)-conjugated secondary antibodies. The experiments were performed independently three times. The images were captured using a Nikon Eclipse Ti-S Microscope, and fluorescence density was quantified using ImageJ. Additional images and quantification of FOXA2 and MUC5AC fluorescence intensities are shown in Figure 3.

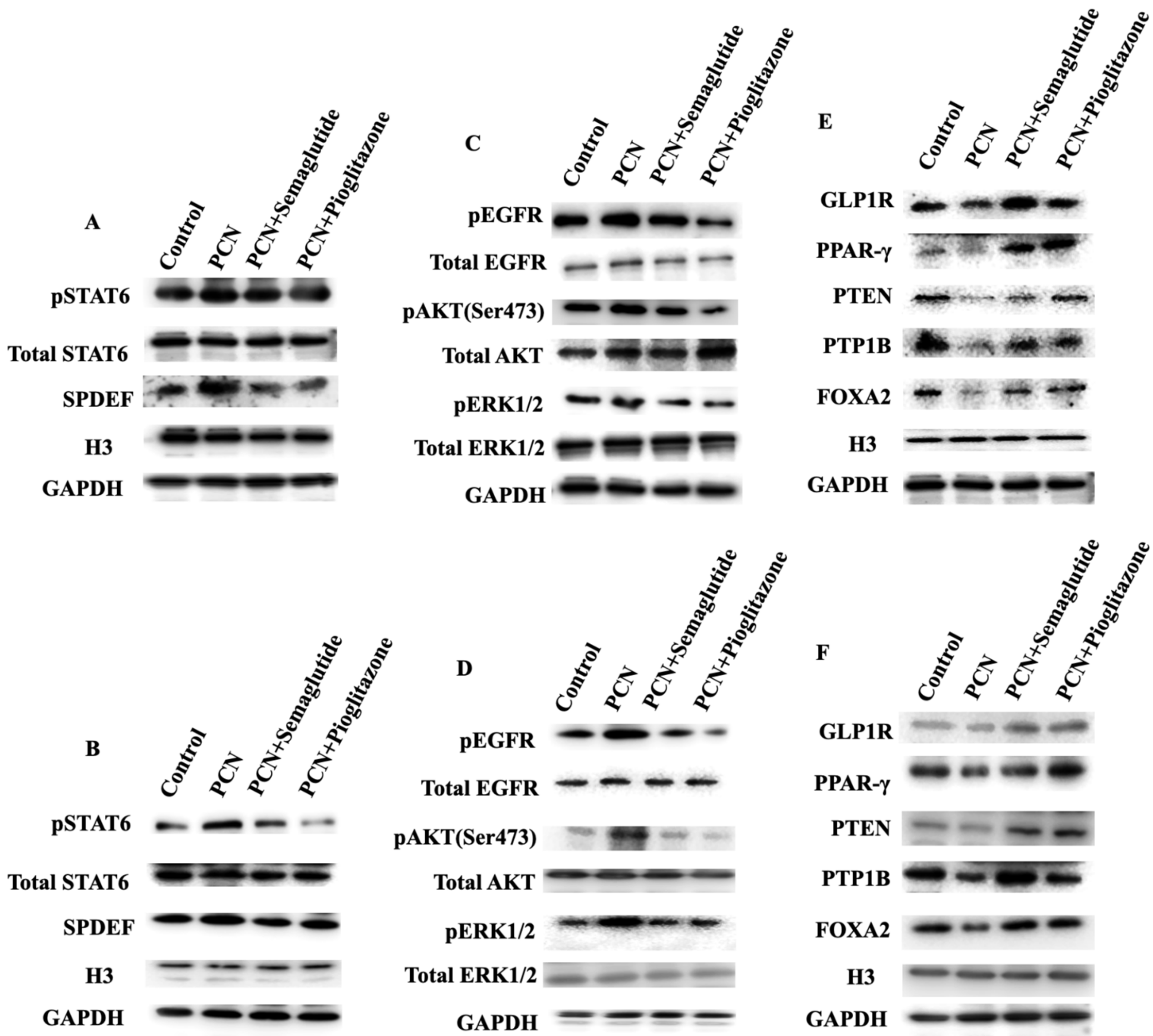

Figure S3. Semaglutide and Pioglitazone attenuate mucin overexpression by restoring the GLP1R-PKA-PPARγ-FOXA2-phosphatase signaling. Submerged 16HBEo- cells were pretreated with Semaglutide (1 μM) and Pioglitazone (10 μM) for 1 hour, followed by exposure to 5 μg/mL PCN for 24 hours. **(A, B)** Semaglutide and Pioglitazone reduce the pSTAT6 and SPDEF expression. **(C, D)** Semaglutide and Pioglitazone markedly inhibit the pEGFR, pAKT, and pERK1/2 expression. **(E, F)** Semaglutide and Pioglitazone restore the expression of GLP1R, PPARγ, FOXA2, and phosphatases PTEN and PTP1B. The experiments were performed independently three times. H3 and GAPDH were used as loading controls. Additional immunoblots and cumulative densitometric analysis are presented in Figure 4.

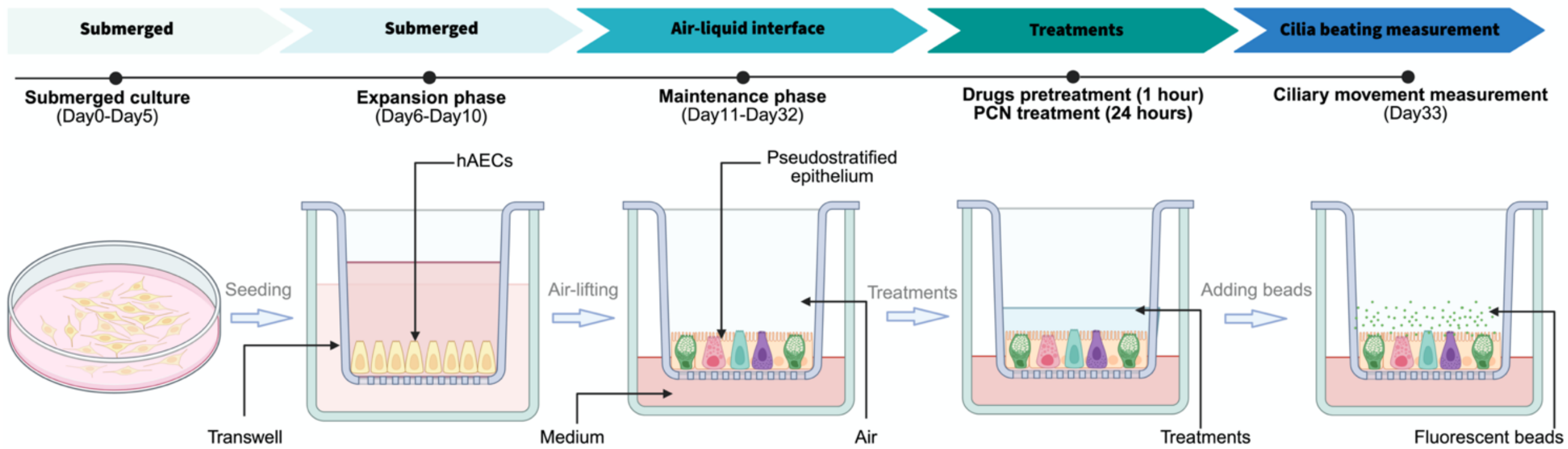

Figure S4. Schemes for ciliary beat frequency (CBF) and ciliary displacement distance (CDD) measurement experiments. Human small airway epithelial cells (SAECs) were seeded on the submerged transwell membrane for 5-7 days, air-lifted, and fed basally with the air-liquid interface (ALI) differentiation medium for 21 days. Then, ALI cultures were pretreated with Semaglutide (1 µM) and Pioglitazone (10 µM) for 1 hour, followed by a 24-hr exposure to 5 µg/mL PCN. Then, the fluorescent beads were added to the apical surface, with motion captured and tracked using a Zeiss LSM 980 microscope. Mucociliary transport rates were measured by reviewing videos and tracking the motion of fluorescent beads, including ciliary beat frequency (CBF) and Ciliary displacement distance (CDD), and analyzed by the Imaris software. The figure was created using the Biorender software (Biorender.com).

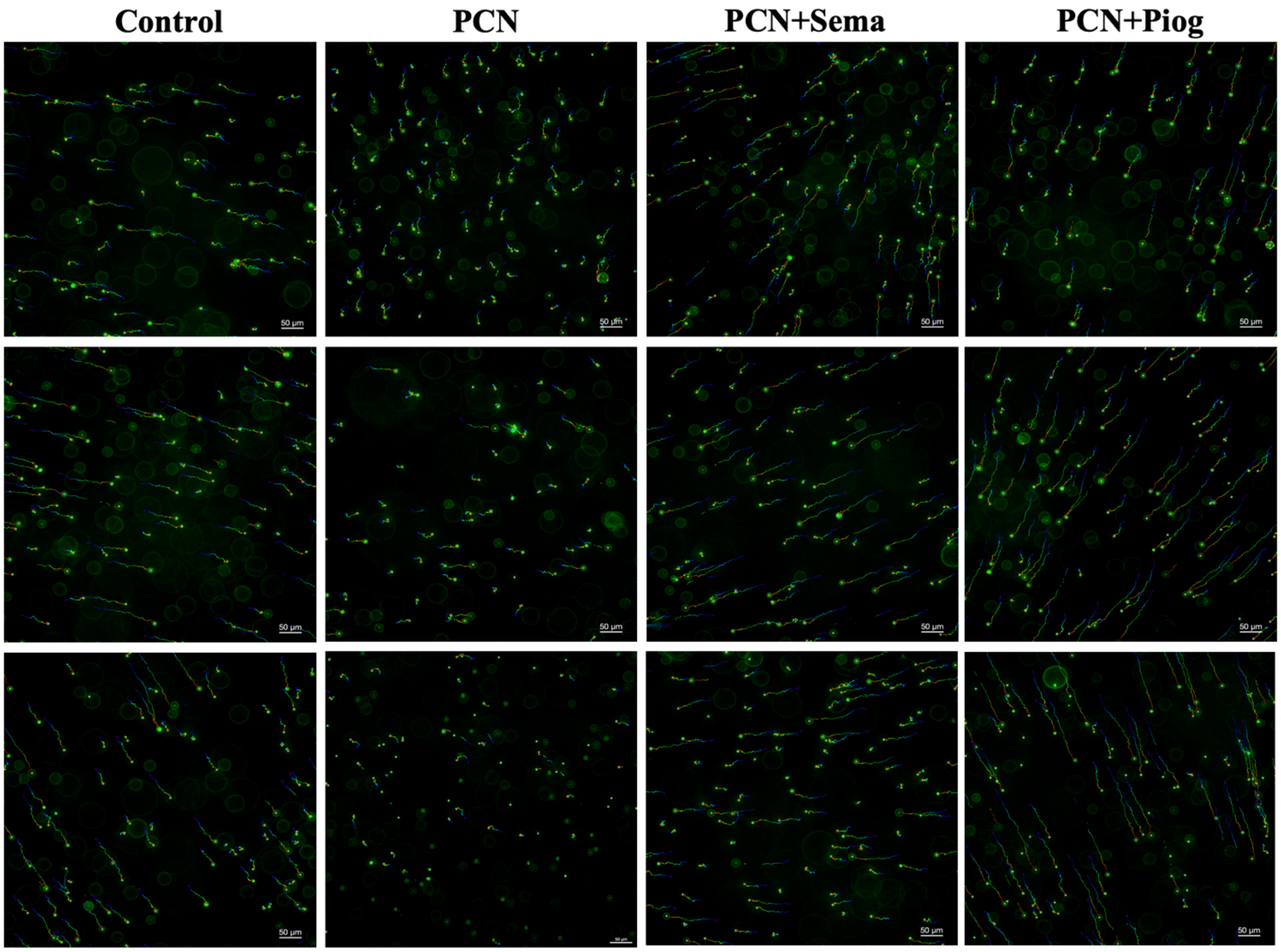

Figure S5. Semaglutide and Pioglitazone restore ciliary beat frequency (CBF) and ciliary displacement distance (CDD) disrupted by pyocyanin. Human primary small airway bronchial epithelial cells (SAECs) were cultured on an air-liquid interface (ALI) as described in Figure S3. ALI cultures were pretreated with Semaglutide (Sema, 1 μM) and Pioglitazone (Piog, 10 μM) for 1 hour, followed by 24 hours of exposure to 5 μg/mL PCN. Then, fluorescent beads were added to the apical surface of the ALI cultures, and CDD and CBF were tracked and recorded using a Zeiss LSM 980 confocal microscope for 1 minute for each sample and further analyzed in Imaris (Oxford Instruments, version 10.2.0) using the Spot module. Images show the tracks of the beads' movement and their distance. The experiments were performed independently twice in triplicates. Additional images and quantification of the CDD and CBF can be found in Figure 5.

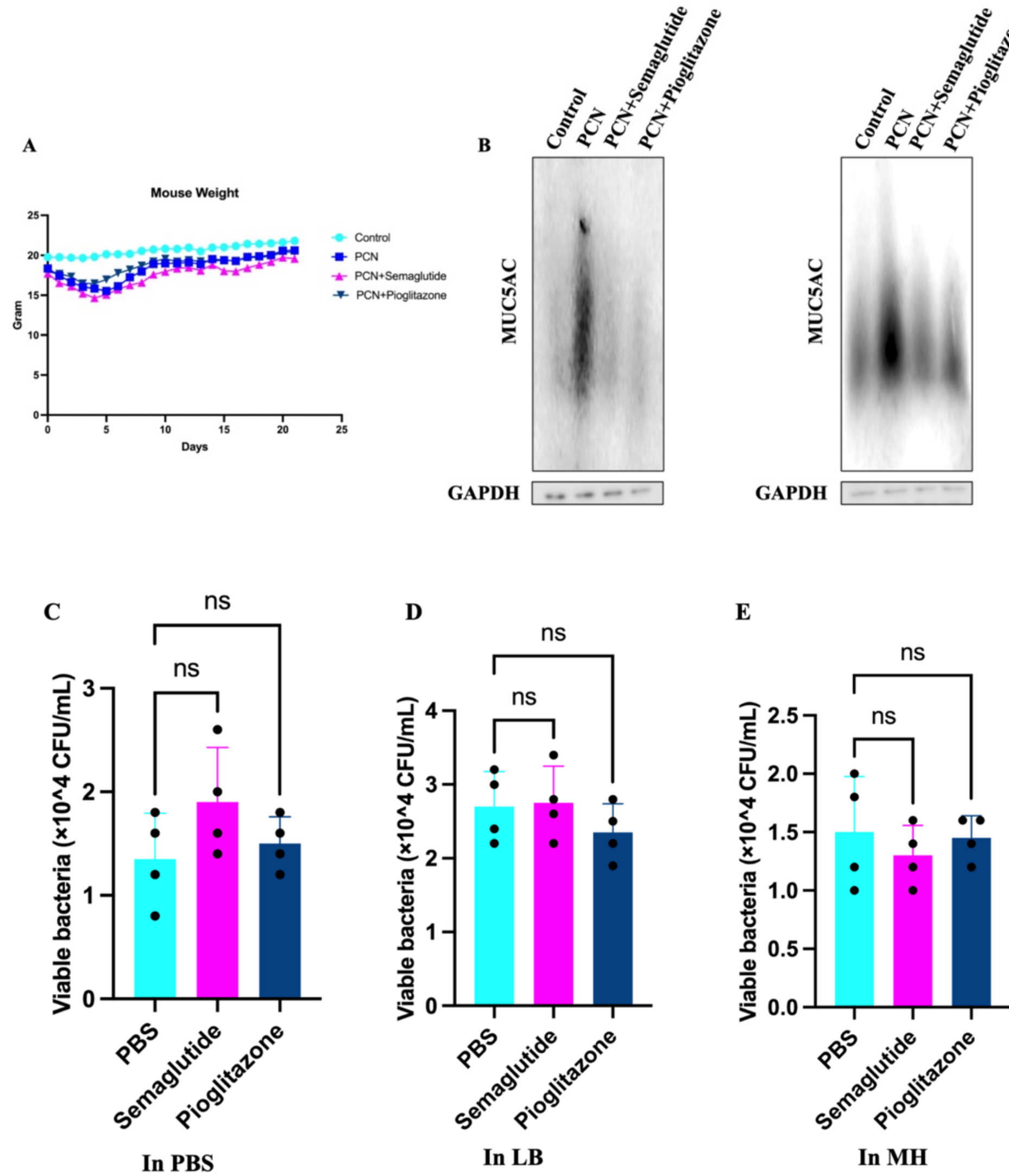

Figure S6. Semaglutide and Pioglitazone attenuate the induction of mucin overexpression by pyocyanin (PCN) and do not directly kill *Pseudomonas aeruginosa*. 6-week-old C57BL6 mice (n = 8) were intranasally exposed to PCN (37.5 µg in 50 µl, once daily) and subcutaneously injected with Semaglutide (160 µg/kg, daily) and Pioglitazone (165 µg/kg, daily) for 2 weeks. **(A)** Mouse weight over the three-week treatment duration. **(B)** The expression of secreted MUC5AC in the mouse bronchoalveolar lavage fluid (BALF) following PCN exposure was analyzed with agarose-acrylamide gels, with GAPDH as the loading control. An additional Western blot is shown in Figure 6C. Densitometry analysis of Western blots in B and Fig. 6C using ImageJ is shown in Fig. 6D. **(C-E)** Semaglutide and Pioglitazone are non-bactericidal. 2.4×10^5 CFU of *P. aeruginosa* strain PAO1 were incubated in PBS, Luria Broth (LB), or Mueller-Hinton (MH) broth and treated immediately with PBS, Semaglutide (1 uM), or Pioglitazone (10 uM), respectively, for 180 minutes. Bacterial colony forming units were determined after serial dilution plating on the Pseudomonas Isolation Agar plates. All data are presented as mean ± s.e.m. ns = non-significant as determined by one-way ANOVA followed by Tukey's multiple comparisons test.

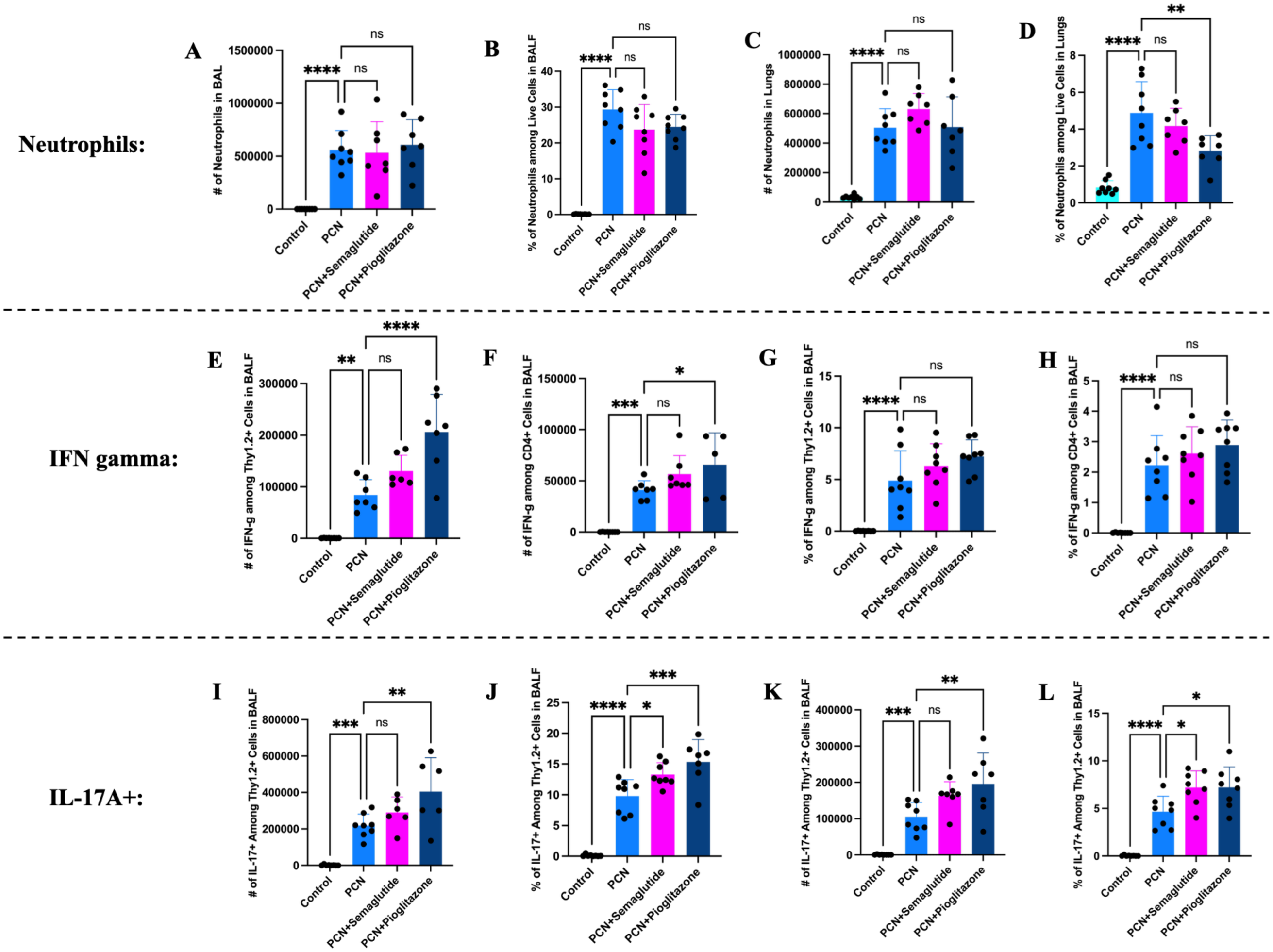

Figure S7. Semaglutide and Pioglitazone do not modulate Th1 and Th17 pulmonary immune responses in a chronic pyocyanin-exposed mouse model. C57BL/6 mice (cohorts of 8, sex-matched) were subcutaneously injected once daily with Semaglutide (160 µg/kg) and Pioglitazone (165 µg/kg), followed by intranasal instillation of 37.5 µg of PCN (in 50 µl) once daily. Control cohorts were subcutaneously injected with 50 µl sterile PBS. After two weeks, mice were euthanized, and bronchoalveolar lavage fluid (BALF) was collected for immune cell analysis. **(A–D)** Quantification of neutrophils, shown as the absolute number of neutrophils in BALF (A), percentage of neutrophils among live BALF cells (B), absolute number of neutrophils in lung tissue (C), and percentage of neutrophils among live lung cells (D). **(E–H)** IFN-γ-producing cells in BALF, shown as the absolute number and percentage of IFN-γ^+ cells among Thy1.2$^+$ cells (E, G) and the absolute number and percentage of IFN-γ^+ cells among CD4$^+$ T cells (F, H). **(I–L)** IL-17A-producing cells in BALF, shown as the absolute number and percentage of IL-17A$^+$ cells among Thy1.2$^+$ cells (I, K) and the absolute number and percentage of IL-17A$^+$ cells among CD4$^+$ T cells (J, L). All data are presented as mean ± s.e.m, and outliers were removed using GraphPad Prism. *p < 0.05, **p < 0.01, ***p < 0.001, ****p < 0.0001 as analyzed by one-way ANOVA followed by Tukey's multiple comparisons test.

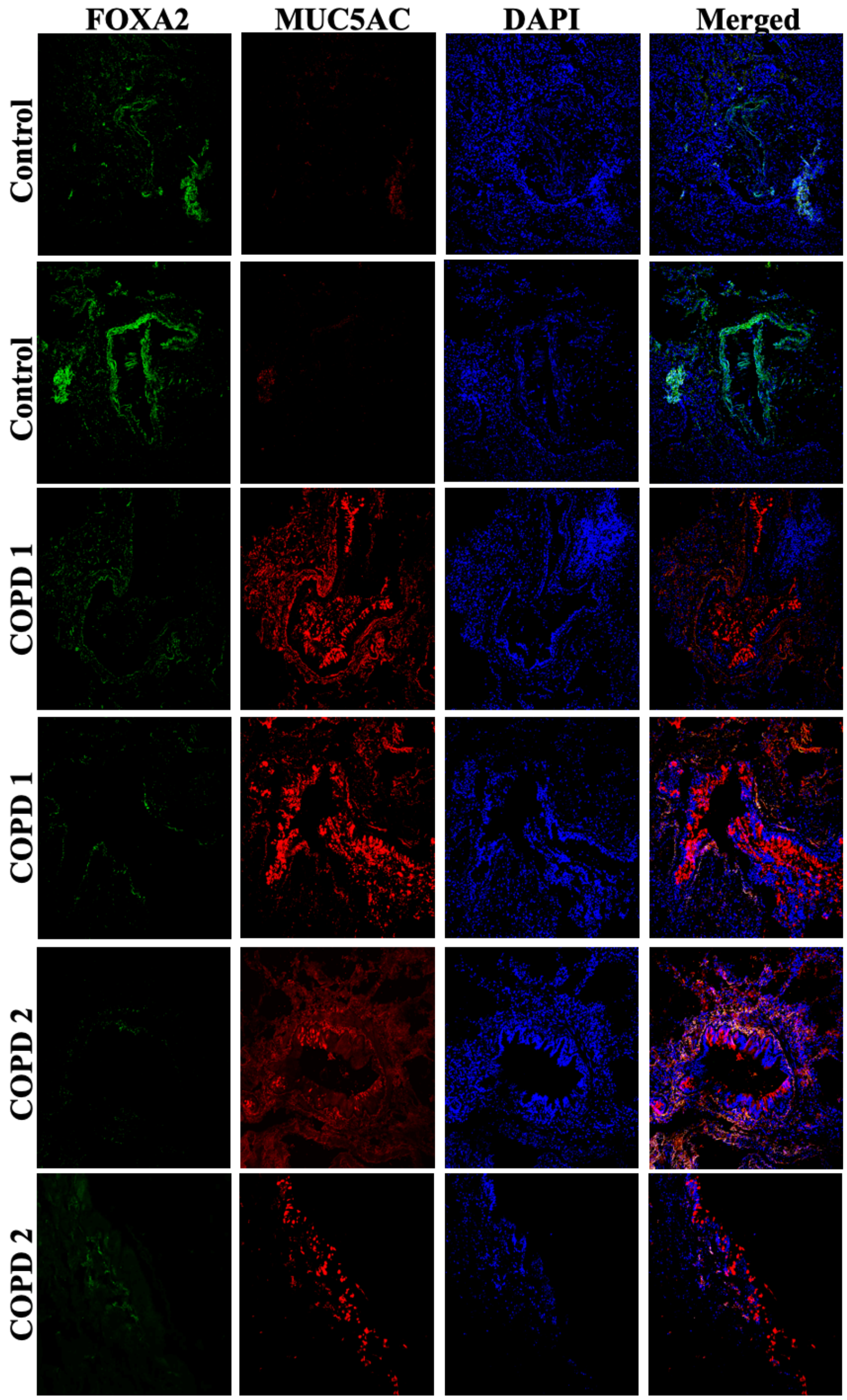

Figure S8. FOXA2 expression is depleted in mucus-overproducing regions of human COPD airways. Representative lung sections from de-identified control donors and patients with COPD were analyzed by immunofluorescence. Tissue sections were co-stained for FOXA2 (green) and the goblet cell–associated mucin marker MUC5AC (red), with nuclei counterstained using DAPI (blue). Merged images demonstrate the spatial relationship between FOXA2 expression and mucus-producing epithelial cells. In healthy control airways, FOXA2 is broadly expressed throughout the airway epithelium, with minimal MUC5AC. In contrast, COPD airways display increased MUC5AC expression accompanied by a marked reduction or absence of FOXA2 in mucus-producing epithelial regions. Images were acquired at 200× magnification. Additional images and quantification are shown in Figure 9.
